# Small-Magnitude Effect Sizes in Epigenetic End Points are Important in Children’s Environmental Health Studies: The Children’s Environmental Health and Disease Prevention Research Center’s Epigenetics Working Group

**DOI:** 10.1289/EHP595

**Published:** 2017-03-31

**Authors:** Carrie V. Breton, Carmen J. Marsit, Elaine Faustman, Kari Nadeau, Jaclyn M. Goodrich, Dana C. Dolinoy, Julie Herbstman, Nina Holland, Janine M. LaSalle, Rebecca Schmidt, Paul Yousefi, Frederica Perera, Bonnie R. Joubert, Joseph Wiemels, Michele Taylor, Ivana V. Yang, Rui Chen, Kinjal M. Hew, Deborah M. Hussey Freeland, Rachel Miller, Susan K. Murphy

**Affiliations:** 1 University of Southern California, Los Angeles, California, USA; 2 Emory University, Atlanta, Georgia, USA; 3 University of Washington, Seattle, Washington, USA; 4 Stanford University, Palo Alto, California, USA; 5 University of California, Berkeley, Berkeley, California, USA; 6 University of Michigan, Ann Arbor, Michigan, USA; 7 Columbia University, New York, New York, USA; 8 University of California, Davis, Davis, Califronia, USA; 9 National Institute of Environmental Health Sciences (NIEHS), National Institutes of Health (NIH), Department of Health and Human Services (DHHS), Research Triangle Park, North Carolina; 10 University of California at San Francisco, San Francisco, California, USA; 11 Duke University, Durham, North Carolina, USA; 12 University of Colorado, Denver, Colorado, USA; 13 National Jewish Health, Denver, Colorado, USA

## Abstract

**Background:**

Characterization of the epigenome is a primary interest for children’s environmental health researchers studying the environmental influences on human populations, particularly those studying the role of pregnancy and early-life exposures on later-in-life health outcomes.

**Objectives:**

Our objective was to consider the state of the science in environmental epigenetics research and to focus on DNA methylation and the collective observations of many studies being conducted within the Children’s Environmental Health and Disease Prevention Research Centers, as they relate to the Developmental Origins of Health and Disease (DOHaD) hypothesis.

**Methods:**

We address the current laboratory and statistical tools available for epigenetic analyses, discuss methods for validation and interpretation of findings, particularly when magnitudes of effect are small, question the functional relevance of findings, and discuss the future for environmental epigenetics research.

**Discussion:**

A common finding in environmental epigenetic studies is the small-magnitude epigenetic effect sizes that result from such exposures. Although it is reasonable and necessary that we question the relevance of such small effects, we present examples in which small effects persist and have been replicated across populations and across time. We encourage a critical discourse on the interpretation of such small changes and further research on their functional relevance for children’s health.

**Conclusion:**

The dynamic nature of the epigenome will require an emphasis on future longitudinal studies in which the epigenome is profiled over time, over changing environmental exposures, and over generations to better understand the multiple ways in which the epigenome may respond to environmental stimuli.

**Citation:**

Breton CV, Marsit CJ, Faustman E, Nadeau K, Goodrich JM, Dolinoy DC, Herbstman J, Holland N, LaSalle JM, Schmidt R, Yousefi P, Perera F, Joubert BR, Wiemels J, Taylor M, Yang IV, Chen R, Hew KM, Freeland DM, Miller R, Murphy SK. 2017. Small-magnitude effect sizes in epigenetic end points are important in children’s environmental health studies: the Children’s Environmental Health and Disease Prevention Research Center’s Epigenetics Working Group. Environ Health Perspect 125:–526; http://dx.doi.org/10.1289/EHP595

## Introduction

Epigenetics is defined as the mechanisms by which mitotically heritable perpetuation of gene activity occurs without modification of the underlying gene sequence. The most commonly studied epigenetic mechanisms are methylation of DNA cytosine residues and the post-translational modification of histone proteins. The entirety of the epigenetic features of the genome are referred to as the epigenome. This layer of regulatory information is essential for proper development of cellular function and determination of cellular identity. Unlike the genome, the epigenome is variable by cell, tissue type, and developmental stage. These mechanisms also represent an adaptive intermediary that interprets and responds to environmental stimuli, resulting in alterations in gene expression. Thus, epigenetic and epigenomic characterization has rapidly become a primary interest for children’s environmental health researchers studying the influence of the environment on human populations, particularly exposures during pregnancy and early life and their impact on childhood and later-in-life health and disease outcomes. Indeed, extensive human epidemiological and animal model data indicate that environmental influences such as stress (Vidal et al. 2014), socioeconomic status (Olden et al. 2014), and exposures to various environmental factors including toxicants (e.g., lead, arsenic, mercury, bisphenol A, cigarette smoke) (Cardenas et al. 2015; Goodrich et al. 2015; Joubert et al. 2012; Koestler et al. 2013; Nahar et al. 2014), nutritional factors (Hoyo et al. 2011; Steegers-Theunissen et al. 2009), parental body mass index (Liu et al. 2014; Soubry et al. 2013, 2015), gestational diabetes (Finer et al. 2015), and maternal antibiotic use (Vidal et al. 2013) during critical periods of prenatal and postnatal development influence developmental trajectories, thereby imparting permanent changes in phenotypic expression of the genome and chronic disease susceptibility.

**Figure 1:**
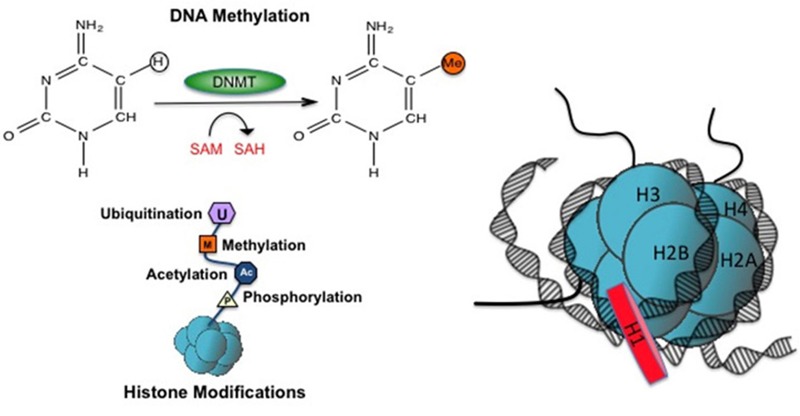
Two major epigenetic modifications. DNA methylation involves the transfer of a methyl group from *S*-adenosylhomocysteine to the 5´ position of the cytosine ring, most often on cytosines followed by guanines in the DNA sequence. This results in the formation of 5-methylcytosine. Histone modifications are another major type of epigenetic modification, and involve the post-translational transfer of, for example, methyl, acetyl, ubiquitin, or phosphate groups to specific amino acid residues on the N-terminal tail of the histone proteins. The N-terminal tails protrude from the center of the nucleosome core (shown on right) and are accessible for these types of modifications. A linker histone (H1) is bound to DNA outside the nucleosome and is thought to help keep the DNA correctly positioned in relation to the nucleosome core.

DNA methylation is the most intensively studied epigenetic modification. It involves the covalent addition of a methyl group (-CH_3_) to the 5´ carbon of a cytosine moiety, generating 5-methylcytosine (5-mC) (Figure 1), which occurs predominantly in the context of cytosines that precede guanines (5´-CpG-3´ dinucleotides, or CpGs). Hydroxymethylation, in which a hydroxymethyl group replaces the hydrogen atom at the 5´ carbon position in cytosine, is a closely related derivative that was conventionally thought to be an intermediate product during 5-methylcytosine demethylation but may also have a role in gene regulation (Hahn et al. 2014; Shen et al. 2014). CpGs are highly underrepresented in the genome, yet an average of 70% of these are methylated in most tissues. The remainder are unmethylated, often found in “CpG islands” that exist throughout the genome and are often present at the 5´ promoter and/or exon region of genes. Nearly 60% of human promoters are characterized by a high CpG content. However, CpG density alone does not influence gene expression. Instead, regulation of transcription often depends on DNA methylation status. In general, promoter-associated CpG islands are unmethylated at transcriptionally active genes, whereas promoter methylation is typically associated with gene silencing. In contrast, intragenic methylation is often positively associated with gene transcription. Thus the impact of DNA methylation on gene activity can vary dramatically depending on context.

**Figure 2:**
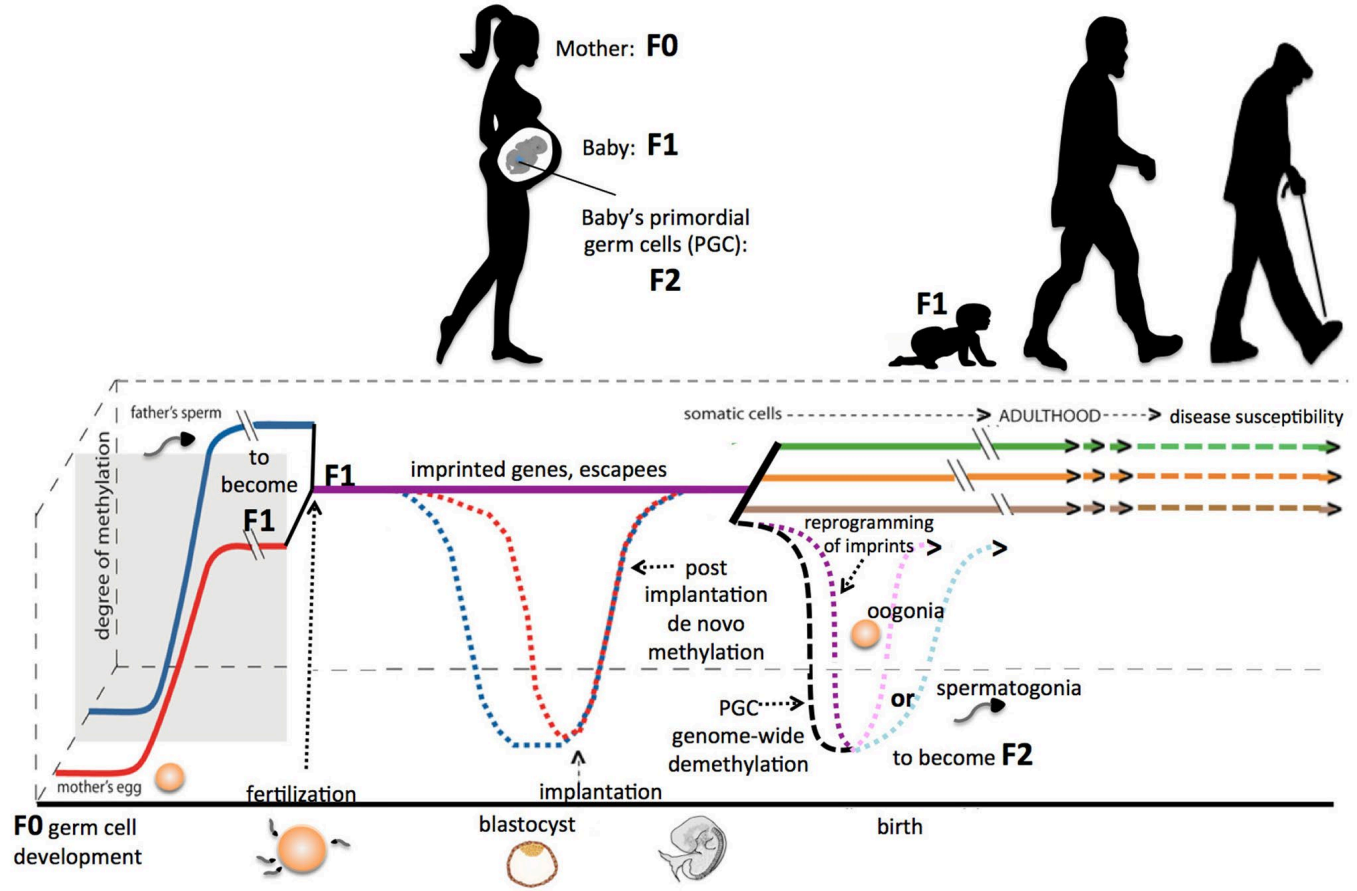
DNA methylation dynamics throughout the human life span. During gametogenesis, the DNA methylation is erased in the primordial germ cells (PGCs) and then acquires new methylation profiles that are in large part sex-dependent, including the methylation present at imprinted genes. At fertilization, the parental pronuclei are erased of nearly all methylation (imprinted genes and “escapees” resist this demethylation—see text). Around the time of implantation, new DNA methylation information is established on the diploid chromosomes in a manner that will aid differentiation of cells to become trophoblast versus embryonic tissues, formation of the three germ layers and then differentiation into the somatic tissues. Many scientists believe that the highly dynamic nature of the genome-wide methylation profiles during these reprogramming and rapid growth periods of development represent windows of vulnerability where an environmental exposure could cause detrimental shifts in methylation by disrupting the fidelity of these reprogramming processes.

Compelling epidemiological evidence of a link between early-life exposure and later disease has been reported (Barker 1988, 1995; Barker and Osmond 1988; Barker et al. 1989; Hales et al. 1991; Leon et al. 1998). Environmental influences that can disrupt development include nutritional factors, endocrine-disrupting agents as well as physiological and psychological stressors. Embryonic and fetal development requires the well-orchestrated formation of key structures. This is carried out in part by the epigenetic modifications that are established during two major epigenetic reprogramming events (Figure 2). The first occurs during gametogenesis, when the vast majority of the DNA methylation information is erased and then reestablished. The second occurs postfertilization when the paternal genome is rapidly erased of most DNA methylation marks followed by erasure of the maternal methylation information. New DNA methylation is established around the time of implantation, before germ layer specification. An exposure that occurs during pregnancy has the capacity to affect three generations at one time, including the mother (F_0_), the developing child (F_1_), and the developing gametes within the developing embryo/fetus (F_2_), which undergo reprogramming in humans from about 4 to 12 weeks gestation. There are regions of the genome that are able to resist postfertilization reprogramming, including imprinted genes (a group of monoallelically expressed genes defined by parent-of-origin dependent methylation and expression), some repetitive elements, and the recently identified group of genes referred to as “escapees” that carry DNA methylation information forward from the prior generation (Tang et al. 2015). Perturbations during these critical developmental windows can lead to responses that likely result in irreversible changes to tissue structure and function (e.g., altered cell type, number and function). In turn, these changes can manifest later in life and have the capacity to modulate physiological function and susceptibility to disease. Research also is emerging that investigates the placenta as a target tissue by which to study exposures at the maternal–fetal interface (Li Q et al. 2015; Maccani and Maccani 2015; Paquette et al. 2015; Schroeder and LaSalle 2013).

A common finding in environmental epigenetic studies is the small-magnitude epigenetic effect sizes that are associated with exposure. It is reasonable and necessary that we question the relevance of such small effect sizes. What is the functional consequence, and do these small differences become magnified over the course of our lives, raising risk for cellular malfunction and disease? It may be the case that we do not find larger effect sizes (e.g., as observed in cancer) not because they do not exist—but rather because such large shifts may be incompatible with continued development. We also must consider the literal meaning of “small” effect sizes. A small difference in DNA methylation, for example, is small only in the context of the population of cells examined as a whole. In any given somatic cell, the autosomes are diploid, which means at any given CpG site, methylation is either present or absent on that chromosome. Within a cell, each autosomal CpG dinucleotide is thus 0% methylated, 50% methylated, or 100% methylated when accounting for the diploid state of the chromosomes. A small difference in methylation means that a small fraction of the cells exhibits this difference at a particular CpG. Depending on the nature and identity of that cell, such a difference could substantially affect that cell’s function and, because of mitotic heritability of DNA methylation, the function of that cell’s progeny.

Here we focus on the epigenetics and epigenomics research being conducted within the Children’s Environmental Health and Disease Prevention Research Centers, or Children’s Centers, as it relates to the “Developmental Origins of Health and Disease (DOHaD)” hypothesis (Barker 1995), which proposes that adverse events during early life program an increased risk for numerous adult diseases. Our objective is to discuss the state of the science in environmental epigenetics research and, in particular, to focus on the collective observations of many studies published thus far that for nearly any given exposure, the magnitude of effect on DNA methylation is relatively small. We will address the current laboratory and statistical tools available for epigenetic analyses, discuss methods for validation and interpretation of findings, particularly when effect sizes are small, question the functional relevance of findings, and discuss the future for environmental epigenetics research.

## Technological Tools Available for Assaying DNA Methylation

### Targeted CpG Measurement

Because DNA methylation (5mC) does not change the detectable sequence of DNA, genetic methods to assay DNA methylation have relied on variations of three basic approaches: bisulfite conversion, methyl-sensitive restriction enzymatic digestion, or 5mC antibody detection or enrichment. Treatment of DNA with sodium bisulfite causes the deamination of cytosine to uracil, but 5-methylcytosine is protected from deamination. Any cytosines detected in the DNA sequence after conversion were methylated in the original sequence. Methyl-sensitive restriction enzymes are those that can cut when the recognition site is either methylated or unmethylated depending on the enzyme, and are most effective when paired with an isoschizomer (a restriction endonuclease that recognizes the same sequence), such as *HpaII* and *MspI*, respectively. 5mC antibody detection or enrichment methods rely on the specificity of monoclonal antibodies to 5mC. Although all methods are effective at discriminating methylation differences using a variety of downstream targeted assays, restriction enzyme-based approaches have a disadvantage in being limited only to assay sites recognized by the enzymes used (5–6% of total methylated CpGs), though this may be tempered somewhat by the ability to combine different enzymes to expand coverage. Antibody-based methods rely on enrichment of methylated DNA, so are less quantitative and specific to individual CpG sites than bisulfite conversion or enzyme-based approaches (Laird 2010).

For targeted gene loci of interest, bisulfite treatment of DNA is followed by polymerase chain reaction (PCR) amplification using primers designed to recognize the converted sequence. Using the traditional Sanger sequencing method, PCR products are cloned and individual alleles sequenced. Pyrosequencing (PSQ) is a “sequencing by synthesis” platform that can quantify the proportion of individual nucleotides at a given position in a sequence [e.g., single-nucleotide polymorphisms (SNPs) or, relevant herein, cytosine versus thymine], providing the ability to detect small differences in methylation among samples or groups due to much greater depth of coverage than Sanger sequencing (Tost and Gut 2007). EpiTYPER offers a similar depth advantage for quantifying sequence mixtures, but instead uses a base-specific cleavage and matrix-assisted later desorption/ionization time-of-flight mass spectrometry (MALDI-TOF MS) approach (Thompson et al. 2009).

### Assessment of Global DNA Methylation

For assessing the impact of environmental exposures relevant to children, a global assessment of total levels of DNA methylation is often desired. The major challenge to the field is that most of the global DNA methylation assays have not been compared for accuracy with a more gold-standard approach such as bisulfite sequencing, and thus may be influenced by a variety of reagent or amplification biases (Laird 2010). A recent community-based benchmarking study of DNA methylation assays concluded that global DNA methylation assays showed lower correlations with each other compared to methods for absolute methylation detection of targeted regions (Bock et al. 2010). High-performance liquid chromatography (HPLC) tandem mass spectrometry (LC-MS/MS) can accurately compare total 5mC with total cytosine in a sample, but it requires large amounts of DNA and may be a less sensitive method than other approaches (Lisanti et al. 2013). Analysis of common repetitive sequences such as LINE-1 by bisulfite treatment and PSQ is one of the most common methods for clinical or epidemiologic samples. PSQ of Alu repeats also has been performed, but the global methylation levels are much lower than those of LINE-1 or genome-wide sequencing, suggesting that complexity of sequence variation of this repeat or the evolutionary context is influencing methylation results (Lisanti et al. 2013; Nelson et al. 2011). LUMA uses a methyl-sensitive restriction digestion followed by PSQ, but was found to be less accurate than LINE-1 or LC-MS/MS on the same samples (Lisanti et al. 2013).

### Genome-Scale Approaches

Microarrays have long been the method of choice for profiling epigenetic marks on a genomic scale, with several platforms and protocols available for DNA methylation (Schones and Zhao 2008). Many of the early platforms used restriction enzyme digests and methylated DNA immunoprecipitation (MeDIP) with an anti-methylcytosine antibody to identify regions of differential methylation by hybridization to oligonucleotide arrays produced in house and by companies such as Agilent and Nimblegen. These include Comprehensive High-throughput Arrays for Relative Methylation (CHARM), in which restriction enzyme McrBC is used to cut methylated DNA and compare to the uncut input DNA (methylated plus unmethylated), among others (Ladd-Acosta et al. 2010). These approaches have resolution sufficient to detect regions of differential methylation and have been used successfully in studies of target tissue in which exposure or disease produced substantial methylation differences among experimental groups (Irizarry et al. 2009; Ji et al. 2010). The coverage of genomic elements (e.g., promoters, gene bodies, CpG islands, shores) depends on the density of probes present on the platform used.

More recently, Illumina developed arrays that allow assessment of single CpG sites, as opposed to regions, at a more quantitative level using bisulfite conversion enabling absolute quantification of methylation levels and detection of small exposure- or disease-associated methylation differences both in target and surrogate tissues (Breton et al. 2009; Morales et al. 2012). The first Illumina 27k array provided coverage for only CpG islands in the human genome, whereas the newer Illumina Infinium HumanMethylation450 BeadChip (“450K array”) provided comprehensive coverage for 99% of Refseq genes with 20 probes per gene on average covering both promoter and gene body as well as CpG islands in the genome (5 probes on average), CpG island shores (5 probes on average), and more distant CpG motifs such as CpG shelves (4 probes on average). This has been the most commonly used platform for genomic analysis of DNA methylation in human cohorts and is especially advantageous for children’s studies with limited samples, because only 250 ng DNA per sample is needed. However, this platform is not available for model organisms commonly used in epigenetic research including mice. In early 2016, Illumina replaced the 450K array with the Infinium MethylationEPIC (EPIC) array which retains > 90% of the original probe content while adding 350,000 CpGs in enhancer regions to improve detection of differential methylation at > 850,000 methylation sites and still requiring only 250 ng DNA per sample (Moran et al. 2016).

Next-generation sequencing technologies are alternative and increasingly used platforms for genomic assessment of altered methylation (Plongthongkum et al. 2014). They include methods that detect regions of differential methylation based on peak finding such as the sequencing analog of MeDIP (MeDIP-seq), Methylation-sensitive Restriction Enzyme sequencing (MRE-seq), and Methyl-CpG Binding Domain (MBD) protein-enriched genome sequencing (MBD-seq). Similar to analogous array-based technologies, these platforms enable detection of more pronounced methylation differences at a level of a region. More quantitative approaches rely on bisulfite conversion and include reduced-representation bisulfite sequencing (RRBS) (Boyle et al. 2012) in which MspI digestion is used to enrich for the most CpG-rich regions of the genome. Also, target enrichment methods based on hybdridization to oligonucleotides interrogate the most informative areas of the genome, regardless of their CpG density. Both RRBS and hybridization-based target enrichment approaches allow for assessment of absolute levels of DNA methylation at each CpG site and for detection of small methylation changes. However, RRBS coverage is restricted mostly to CpG islands, and coverage varies between individual samples. Hybridization-based capture approaches can be customized to target genes or regions of interest, but this approach showed lower reproducibility compared with amplicon-based bisulfite sequencing of targeted regions. Whole-genome bisulfite sequencing (WBGS) techniques have not been used widely in exposure and disease studies in human cohorts and animal models due to the expense and the complexity involved in the analysis of such large data sets. However, for most epidemiology studies high coverage of individual CpG sites is not required, and indexed sequencing libraries from 100 ng of DNA can achieve depth of 0.2× to 3× coverage at a fraction of the cost, and represent the most unbiased representation of CpGs in the genome. AmpliconBS, in which 10–20 targeted PCR amplicons from bisulfite DNA are pooled and sequenced, outperformed most other absolute targeted DNA methylation assays in a community-based benchmarking study (Bock et al. 2010).

At the present time, however, most publicly available data sets have been collected on the Illumina 450K array platform, and analysis methods for this platform have reached maturity (Aryee et al. 2014), whereas those for sequencing-based approaches are still under development (Plongthongkum et al. 2014). Using this platform therefore offers a great advantage of easy comparison across different studies and relatively broad availability of published studies for validation purposes.

## Integrative Data Analysis for DNA Methylation in Birth Cohort Studies: Challenges of Data Processing and Statistical Analysis

Early-life exposures typically produce relatively small effects on DNA methylation. Thus, maximizing data reliability via stringent quality control and data processing procedures, as well as statistical power to detect small-scale changes, is crucial for identifying environmental epigenetic links. Here we discuss these principles with regard to birth cohort and other longitudinal children’s studies evaluating environmental factors as they apply to two widely used bisulfite-treatment methodologies: *a*) quantitative targeted DNA methylation analysis by PSQ and *b*) epigenome-wide analysis with the Infinium 450K or EPIC array [we refer readers to recent publications that provide more detail on specific aspects of the 450K array pipeline, data processing, and analysis (Heiss and Brenner 2015; Maksimovic et al. 2015; Morris and Beck 2015; Robinson et al. 2014; Yuan et al. 2015)].

Approaches to analyze DNA methylation data from birth cohorts or other longitudinal children’s cohorts fall into three broad categories based on the timing of available data and the hypotheses: *a*) cross-sectional, *b*) longitudinal, and *c*) mediational analyses. Longitudinal analysis is optimal to assess the impacts of early-life and concurrent exposures on DNA methylation and intra-individual variability in DNA methylation “drift” over time (Issa 2014). The ultimate goal is to assess whether epigenetic change acts as a mediator between environment and outcome (e.g., *in utero* exposure and altered childhood growth trajectory). Linear regression and structural equation modeling are both commonly used for mediational analysis (Baron and Kenny 1986; Li 2011). Scale restriction makes detailed assessment of all interrogated CpG sites within a region or across the genome as mediators difficult. Thus, first applying dimension reduction methods such as principal component analysis (Lam et al. 2012) to the data can help investigators select a smaller number of variables to represent methylation at key regions in mediational analysis. When analyzing DNA methylation data to address hypotheses in any of the three categories, the nature of DNA methylation data—both continuous and finite with a beta distribution—must be considered. Variance stabilizing transformations should be considered to avoid violating the assumption of constant variance in normal regression, and beta regression should be used when DNA methylation is not normally distributed.

### Key Covariates for DNA Methylation Analysis

Regardless of the source of DNA methylation data or type of analysis, covariates and confounders to consider when assessing relationships between environmental factors and DNA methylation in neonatal samples or childhood samples minimally include gestational age, sex, maternal smoking status, socioeconomic status, and race (Goodrich et al. 2015; Joubert et al. 2012; Murphy et al. 2012; Vilahur et al. 2014; Yousefi et al. 2015a). Given sex differences observed in DNA methylation and response to environmental exposures, sex-stratified analyses or examination of sex–exposure interactions are also worthwhile statistical pursuits when sample size allows (Murphy et al. 2012; Vilahur et al. 2014).

Common source tissues for DNA collected in neonatal and children’s studies (e.g., placenta, buccal, blood, saliva) are heterogeneous with regard to cell type composition. Several studies have demonstrated that the degree of DNA methylation at specific loci is dependent on the type of tissue under examination (Davies et al. 2012; De Bustos et al. 2009; Lowe et al. 2015), and this variation can exceed the variation across individuals (Lokk et al. 2014). Cell-type heterogeneity within tissues can confound statistical analyses when cellular composition between controls and cases is divergent. Thus, when DNA is not obtained from sorted cells, adjustment for cell-type percentages in the main model or in subsequent sensitivity analyses will increase the reliability of associative findings whenever differential counts are available (Burris et al. 2013; Huen et al. 2014; Tarantini et al. 2013; Yousefi et al. 2015b). This is especially important in children’s environmental health research because some exposures (e.g., arsenic) and age can affect both DNA methylation (Koestler et al. 2013; Yuan et al. 2015) and cell-type populations (Bellamy et al. 2000; Cheng et al. 2004; Kile et al. 2014). Houseman et al. proposed a method, based on data from a reference sample of isolated purified leukocyte subtypes (Houseman et al. 2012), that has been refined using 450K data available on leukocytes subtypes (Reinius et al. 2012) and more recently using data from cord blood leukocyte subtypes (Bakulski et al. 2016). This method allows for changes in the relative proportions of cells associated with exposure or phenotype to be assessed by estimating the proportion of individual cell types, and this could provide important insights into the true effects of exposures on children’s health outcomes. The accuracy, reliability, and utility of this estimation from array-based DNA methylation data were subsequently demonstrated in a series of reports (Accomando et al. 2014; Koestler et al. 2013).

As more reference data become available for additional leukocyte types or for various specific cell types from other tissues, potentially from data available through the Roadmap Epigenome Project, these types of estimations could become more widely available. Until that point, Zou et al. (2014) and Houseman et al. (2012, 2014) have developed reference-free methodologies, which use a surrogate variable type approach to control for cellular heterogeneity in the absence of a reference data set, approaches well-suited for environmental epidemiology studies making use of non-blood biological samples for analysis (e.g., placenta). However, the use of reference-free methods assumes that outcome-related changes will be larger than cell type–specific changes, which may not always be the case.

### Statistical Model Selection for Targeted DNA Methylation Analysis

Statistical model selection with regard to treatment of individual CpG sites is important when examining associations between exposures and DNA methylation at targeted regions (e.g., PSQ data). In the aforementioned simulation studies, maximum statistical power was achieved when using a generalized linear model (GLM) that treated methylation at CpG sites within the bisulfite sequenced region as repeated measures with unstructured variances and covariances (Goodrich et al. 2015). This modeling strategy has the ability to identify exposure–DNA methylation relationships for the entire region as well as at individual CpG sites with the addition of an interaction term. An alternative modeling strategy that captures both intragenic CpG site-specific differences and variation between technical replicates utilizes linear mixed-effects regression with random effects for sites and replicates (Burris et al. 2012; Huen et al. 2014; Vilahur et al. 2014). The aforementioned models are used primarily for cross-sectional or longitudinal studies with methylation data at a single time point (e.g., prenatal exposure and DNA methylation in childhood). Analysis methods for longitudinal studies with DNA methylation data from multiple time points (e.g., birth and adolescence) include generalized estimating equations (GEE) which treat DNA methylation data from the same individual at different times as a cluster (Hou et al. 2014; Zeger et al. 1988). Mixed-effects models for repeated measures also can be used to examine the association of exposure with methylation at a targeted region (e.g., LINE-1 repetitive elements) from multiple time points (Baccarelli et al. 2009).

### Illumina Infinium HumanMethylation450 and MethylationEPIC BeadChips

**Figure 3:**
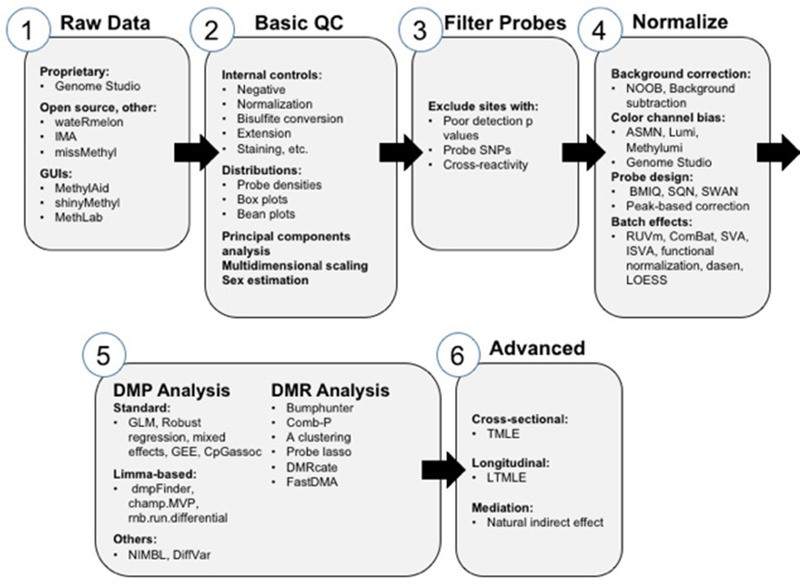
Detailed comparison of 450K preprocessing methods. GUI, graphical user interface. Workflow for analysis of data generated on the HumanMethylation450 BeadChip and options for analysis at the various steps.

Before epidemiological analysis can be performed with 450K or EPIC BeadChip data, as with any data file, it is imperative to perform quality assurance and quality control checks and data preprocessing to ensure that technical variation has been minimized and that remaining observations are free from several common sources of bias. Here we provide a brief overview of the typical steps involved and software offerings available for these preprocessing steps (Figure 3, steps 1–4). All analysis pipelines described here for 450K data can be applied to data from the new EPIC BeadChip. Following preprocessing, all software options can return a matrix of methylation percentages, or β values ranging from unmethylated (0) to completely methylated (1), for all retained samples and CpGs. Analysis can be run using this β scale or can be logit transformed to M-values to avoid heteroscedasticity when modeling (Du et al. 2010).

### 450K Statistical Methods: Linear Models

To date, epidemiological analysis with 450K data has generally relied on linear modeling approaches similar to those for PSQ, only on a larger scale due to the increased number of CpGs interrogated. However, as algorithmic batch effect removal is often performed during 450K preprocessing, explicitly modeling batch as a random effect or additively as a model covariate may not be required. Several methodologies have been proposed for removal of batch effects (Fortin et al. 2014; Heiss and Brenner 2015; Leek and Storey 2007, 2008; Maksimovic et al. 2015; Pidsley et al. 2013; Teschendorff et al. 2011), and ComBat (Johnson et al. 2007; Leek et al. 2012) appears to be one of the most effective. When this is the case, an ordinary GLM can be used in cross-sectional analyses to determine the change in DNA methylation per unit change in an exposure of interest, adjusting for the key covariates explored above. In the longitudinal setting, again standard linear methods such as mixed effects or GEE models are appropriate (Figure 3, step 5).

### 450K Statistical Methods: *limma*-Based Estimators

In addition to ordinary regression performed with standard statistical software, use of the *limma* linear modeling Bioconductor package has become a popular option in 450K data analysis (Smyth 2005). The *limma* package has been incorporated into common 450K analysis pipelines (e.g., the “dmpFinder” function in *minfi* and the “champ.MVP” in *ChAMP*) (Aryee et al. 2014; Morris et al. 2014). The *limma* model allows for stable estimates when performing analysis with small sample sizes (Smyth 2005).

### 450K Statistical Methods: Causal Approaches

The most widely used approach to mediation analysis is the Baron and Kenny framework (Baron and Kenny 1986), which requires a series of regression models to determine whether a variable can be considered a mediator. This approach is hindered by its low power to detect an effect (Fritz and MacKinnon 2007). Further, the presence of mediation is indirectly inferred by looking at the relationship of *a*) the independent variable with the mediator and *b*) the mediator with the dependent variable rather than estimating that actual indirect effect itself (Hayes 2009). Parametric linear models are appealing in the context of array-based DNA methylation data analysis, but it may be preferable to implement semi- or nonparametric models that involve fewer assumptions. Two types of methodologies that have been applied to genomics and epigenomic studies are the Targeted Minimum Loss-Based Estimation (TMLE) (Figure 3, step 6) and Mendelian Randomization.

TMLE is a double robust semiparametric efficient estimation method, and is tailored to minimize bias and maximize precision as proven by theory (Chambaz et al. 2011; Robertson 2005; Tuglus and van der Laan 2011; van der Laan 2010a, 2010b; van der Laan and Rose 2011; van der Laan and Rubin 2006; van der Laan et al. 2009; Wang et al. 2011). TMLE works by using an ensemble machine learning algorithm, SuperLearner (van der Laan and Rose 2011; van der Laan et al. 2007), to obtain an initial estimate of the regression of the outcome on the target variable and the confounders, and then using a targeted bias reduction step that incorporates an estimate of the propensity score. SuperLearner provides a substantial modeling advantage because it uses cross-validation to select the best weighted combination of estimators from a user-defined library of candidate estimators and has been shown to be theoretically and practically superior to any of the individual candidate estimators in the library (van der Laan and Dudoit 2003; van der Vaart et al. 2006). The model library can include as diverse a set of models as can be conceived by the analyst—for example, any flavor of linear model, spline-based techniques (Friedman 1991), regression tree algorithms such as Random Forest (Breiman 2001) or Bayesian Regression Trees (Chipman et al. 2010), or many others could all be used each with many different tuning settings. The TMLE method can readily be implemented using the TMLE R package (Gruber and van der Laan 2012). Additionally, the TMLE theory has recently been optimized to perform similar estimation in the longitudinal setting (Petersen et al. 2014; van der Laan and Gruber 2011), and now a dedicated L-TMLE software package has also been released (Figure 3, step 6) (https://github.com/lendle/tmlecte).

TMLE is an optimal way to perform detailed mediation analysis. The mediating role expected for biological factors such as DNA methylation can be conceptualized as the natural indirect effect (NIE) described in the causal inference literature (Figure 3, step 6) (Lendle et al. 2013; Petersen et al. 2006). Under a counterfactual framework, the NIE is simply the difference between natural direct effect (NDE), or the effect of the exposure on the outcome holding the intermediate variable at what would have been its value at a reference exposure level, and the total effect of the exposure on the outcome. Software to estimate each of these quantities (NIE, NDE, and the total effect) by TMLE has recently been made available in the tmlecte package (https://github.com/lendle/tmlecte).

The Mendelian randomization approach has been utilized in epidemiologic studies as another methodology for estimating causal inference (Davey Smith and Hemani 2014; Relton and Davey Smith 2012, 2015). It relies on use of genetic polymorphisms that are *a*) highly associated with the modifiable intermediate but *b*) not associated with the health outcome of interest. The strength in this approach is that the estimate of the relationship of the highly correlated genetic variant with the outcome of interest is less prone to biases related to unmeasured confounding and reverse causation. Mendelian randomization has also been applied to epigenomic studies (Binder and Michels 2013; Richmond et al. 2016). To study mediation in particular, a two-step process has been described (Relton and Davey Smith 2012). The first step involves identification of a genetic variant that is strongly associated with the environmental exposure of interest (e.g., smoking, phthalates). Next a genetic proxy highly associated with DNA methylation (e.g., CpG site or region) will also be utilized. From there, the causal relationships between the exposure and the intermediate and also the intermediate and outcome can be estimated. Limitations of this approach include the requirement of larger sample sizes and the potential for genetic confounding that can be introduced by population structure (Relton and Davey Smith 2015).

### 450K Statistical Methods: DMRs

As DNA methylation analysis proceeds, researchers have increasingly focused on identifying differentially methylated regions (DMRs), also known as regions of altered methylation. DMRs are of interest for two reasons: *a*) CpG sites are not expected to function independently, but rather in groups to regulate gene expression, and *b*) observed differences in methylation and individual sites are more likely to be believed if neighboring sites show similar changes. Due to the increasing interest, approaches for DMR identification have proliferated in the last few years (Aryee et al. 2014; Butcher and Beck 2015; Jaffe et al. 2012; Pedersen et al. 2012; Peters et al. 2015; Sofer et al. 2013). An overview of currently available methods is shown in Table 1. These fall into two conceptual categories: *a*) those that perform individual CpG analysis first and then combine results into DMR groupings (Aryee et al. 2014; Butcher and Beck 2015; Jaffe et al. 2012; Pedersen et al. 2012; Peters et al. 2015), and *b*) those that group CpGs first and draw inference after the fact (Sofer et al. 2013). In the first group, measures of site-level results (e.g., an effect size or *p*-value) are typically aggregated across genomic coordinates according to smoothing functions, correlation structure, and/or genomic annotation, followed by drawing statistical inference on putative DMRs according to method-specific definitions. The second approach, of which *aclust* is the only current example, applies a clustering algorithm to reduce dimensionality prior to performing statistical tests of association.

**Table 1:** Summary of methods for identifying regions of altered methylation.

Method	Package name	Platform	Analysis order	References
Bump hunter	Minfi	R	Site-first	Aryee et al. 2014; Jaffe et al. 2012
Comb-P	Comb-P	Python	Site-first	Pedersen et al. 2012
FastDMA	FAstDMA	C++/Python	Site-first	Wu et al. 2013
A-clustering	Aclust	R	Cluster-first	Sofer et al. 2013
Probe Lasso	ChAMP	R	Site-first	Butcher and Beck 2015
DMRcate	DMRcate	R	Site-first	Peters et al. 2015

Although several DMR-finding packages exist, this field is still early in its development, and several aspects of method performance require additional characterization. This includes additional validation of the functional impact of identified DMRs in terms of gene expression (Robinson et al. 2014; Yuan et al. 2015). Further, sensitivity analysis on DMR calls has been rare to date. For example, for site-first–type approaches little is known about how effect-size outliers may drive the dimensions of called DMRs. Similarly, the stability and accuracy of DMR boundaries has not been sufficiently evaluated. Another obstacle that all DMR-finding methods must confront is how to appropriately adjust for multiple comparisons, because it is often difficult to determine what constitutes an “independent” test.

DMR finding in the context of longitudinal cohorts, especially those involving infants and children, raises still further considerations. Foremost is the issue of the temporal stability of DMRs called by existing methods. Although much attention has been devoted to age-related changes for individual CpGs, this topic has only just begun to be explored at the level of DMRs in studies involving children (Yuan et al. 2015).

Overall, many of the obstacles faced in developing robust DMR-finding algorithms stem from the lack of a clear definition for DMRs. This can be especially problematic in the sparse-data scenario of array-based DNA methylation analysis where many of the useful data are missing. However, as data from WGBS become increasingly available and DMR functional characterization proliferates, these methods are likely to improve.

## Data Integration and Visualization

Following quality control, data processing, and statistical analyses, visualization of descriptive data and analysis results can be implemented using a variety of approaches. Typically packages in R can be used as well as independent coding or use of general graphics tools. Common useful plots for visualizing DNA methylation data include *a*) pairwise correlation of methylation values across CpGs according to genomic location; *b*) Manhattan plots displaying –log_10_ (*p*-values) from statistical analysis according to genomic location of CpGs; *c*) general heat maps to display correlation of methylation values and/or coefficients from statistical models; and *d*) lollipop-like visualization to compare methylation values across samples, tissues, or other categories. Approaches implemented depend on the type of data analyzed.

R packages that can implement some of all of the above include MethVisual (Zackay and Steinhoff 2010), methyAnalysis (version 1.12.0; R Project for Statistical Computing), Methylation plotter (Mallona et al. 2014), MethTools (Grunau et al. 2000), MethylMix (Gevaert 2015), IMA (Wang et al. 2012), coMET (Martin et al. 2015), and minfi (Aryee et al. 2014) (Table 2). Most of these enable implementation of site-level as well as region-level DNA methylation analysis based on the 450K array including analysis pipeline and processing steps. Although most are implemented with R code, some tools such as coMET and MethTools offer a Shiny web service that can be used as an alternative to the programming method for generating plots, increasing the opportunity for use by researchers working outside of R.

**Table 2:** Example visualization approaches for epigenome-wide DNA methylation data.

Method	Utilities	Implementation	Application/comments
MethVisual	Exploratory data analysis and visualization	R	For bisulfite sequencing data, not genome-wide DNA methylation data (i.e., from Illumina 450K array)
methyAnalysis	Data analysis and visualization	R	For bisulfite sequencing data, not genome-wide DNA methylation data
Methylation plotter	Visualization only	Web	User-friendly, more general descriptive analysis and visualization; more appropriate for small number of samples compared to large sample size of individuals
MethTools	Exploratory data analysis and visualization	R and web	For bisulfite sequencing data, not genome-wide DNA methylation data
MethylMix	Data analysis and some visualization	R	For genome-wide DNA methylation data; implements specific beta mixture model and may not have full flexibility desired
IMA	Data analysis and some visualization	R	For common exploratory analysis of genome-wide DNA methylation data; standard pipeline may limit flexibility
CoMET	Visualization only	R and web	Appropriate for various types DNA methylation data
Minfi	Data analysis and some visualization	R	For genome-wide DNA methylation data; offers fair amount of flexibility
Independent coding	Data analysis and visualization	R	Appropriate for various types of DNA methylation data; specific to analysis and data needs; independent of data input and format requirements of packages but may require more analysis time and skill compared to other methods

## Approaches for Validating/Replicating Loci that Emerge as Top Hits from Primary Analysis

To understand the likelihood that technically and biologically “real” associations have been identified between an environmental exposure and differences in DNA methylation, several approaches for validating or replicating results can be employed. These include technological or platform validation, comparing results with other results published in the literature, replication using a different population, and meta-analysis.

Technological validation typically involves using another platform, such as PSQ if results were originally generated on the 450K, to measure DNA methylation of a handful of CpG sites of interest in the same population in which the original associations were identified. Many individual CpG sites on the 450K array appear to cross-validate well with PSQ (Roessler et al. 2012). Correlation coefficients can then be computed to directly compare the two measurements in the same individuals.

Perhaps the ideal approach for replicating environmental exposure–CpG methylation associations would be to conduct the exact same methylation measurements in a separate yet comparable population with similar measures of environmental exposure. The same statistical modeling approach can be employed in both populations, making direct comparison of results, including magnitudes and direction of effect, feasible. The disadvantages to this approach are the identification of a comparable population, and the time and costs associated with conducting the replication measurements. A good example of this approach is in the paper by Joubert et al. (2012) in which CpG loci associated with maternal smoking were initially identified using the 450K platform in the Norwegian Mother and Child Cohort study (MoBa), and then 26 significant loci were assessed in a separate 450K analysis in the Newborn Epigenetics STudy (NEST). In both cohorts, the platform was the same, methylation was measured in cord blood, exposure was categorized in a similar way (any smoking by the mother during pregnancy), Caucasian/European ancestry participants were included in the analyses (subset of NEST), and the statistical model and covariates were aligned. This approach also has been used in several studies that first identified CpG sites using arrays, and then validated the loci using PSQ (Breton et al. 2009; Devaney et al. 2015; Lazarus et al. 2015).

An alternative approach for large studies is to split the population into a discovery group and a replication group. A question of adequate sample size for the replication study often also arises. For practical considerations, often the replication population is smaller than the original population (Argos et al. 2015; Joubert et al. 2012). However, the proportion exposed should also be taken into account. For example, the NEST population (*n* = 36) used for replication of the MoBa findings included 18 smokers (50% exposed) and 18 nonsmokers (50% unexposed), which enhanced statistical power given the relatively small sample size (Joubert et al. 2012). Although there are no standard guidelines in place when choosing a replication analysis, a strategy that is anticipated to achieve adequate statistical power to detect the observed effect size is warranted. Overall, this approach has been successfully used and greatly enhances the confidence in observed results when the original results are replicated.

Last, in recent years the creation of large consortia in which like datasets are combined in a harmonized fashion to increase the power to detect associations has gained appeal. Several consortia with a focus on epigenetics have been formed including many GWAS (genome-wide association studies) consortia [CHARGE (Childhood Autism Risks from Genetics and the Environment), WHI (Women’s Health Initiative), GIANT (Genetic Investigation of ANthropometric Traits), others], some of which also have DNA methylation data for adults (CHARGE), and newborns and children (PACE). The Pregnancy and Child Epigenetics Consortium (PACE) was created in 2013, and now combines data sets for > 20 cohorts. Recently, a first PACE paper focused on the effects of maternal smoking on the 450K data in the cord blood from 13 participating cohorts has been published (Joubert et al. 2016). It has identified 6,073 loci differentially methylated at genome-wide significance including 2,965 CpGs that are novel—orders of magnitude more loci than identified in any previous study on effects of maternal smoking. Remarkably, it has also replicated most of the main results previously found in individual studies.

Consortium analyses can be extremely powerful in answering a variety of study questions, depending on the availability of exposures and end points measured in the consortium participants. Consortium analyses typically require each study to independently implement a common analysis protocol and provide the results to a central location for meta-analysis. This can accommodate multiple studies, much more than replication analyses, and may be more stable to population heterogeneity, depending on the participants. The ability to accommodate a greater number of studies, increasing sample sizes into the thousands, has substantial impact on statistical power. The approach also promotes data sharing, as often required by the National Institutes of Health (NIH). However, strong coordination and communication across research groups is required to carry out successful meta-analysis, and often requires greater work “up-front” than simpler replication analyses.

Regardless of approach, not all loci will replicate. There are a number of reasons why replication may not be achieved, though it is often difficult to discern the precise reason for any given analysis. Possible reasons for failure to replicate include *a*) the original result was a false positive, *b*) technical or biological differences in the laboratory measurement of DNA methylation introduce a bias or measurement error, or there was *c*) variability in exposure assessment or *d*) differences in the statistical approach between the original and replication analyses. In fact, epigenetics studies may have stricter replication requirements compared with studies with genotyping data (GWAS) due to technical and true variation across study populations. Nevertheless, studies demonstrating lack of replication provide important information (Oliver et al. 2013; Wei et al. 2012), reduce publication bias, and may improve interpretation of complex data.

## Investigating the Functional Relevance of Replicated Loci

### Magnitudes of Effect

The goal of epigenetic studies linking environmental exposures and children’s health is to aid in the understanding of how environmental factors can influence health phenotypes at birth and over the course of a lifetime. Thus, it is important not only to identify valid and replicable variation in DNA methylation or other epigenetic mechanisms with environmental factors or outcomes, but to begin to consider how this variation can be contributing to phenotypes.

Understanding the functional importance of environment-associated DNA methylation variation is challenged by the generally small to moderate differences being observed in relation to various environmental exposures. Initial studies of *in utero* exposure and DNA methylation in offspring focused on repetitive element DNA methylation, as a marker of global DNA methylation status. For example, in a Bangladeshi cohort, comparing the highest to lowest quartiles of maternal urinary arsenic was associated with increased LINE-1 methylation of 1.36% [95% confidence interval (CI): 0.52, 2.21%] (Kile et al. 2012). Among Mexican-American children in rural California, a 1-log increase in maternal serum *o,p´-*DDT levels was associated with a reduced ALU methylation of 0.37% (Huen et al. 2014). Contrast these differences with the reductions that could be observed comparing pathologically normal and tumor tissues, where differences can be 5–20% for LINE-1 (Cho et al. 2010; Matsuda et al. 2012; Stricker et al. 2012; Zhang et al. 2012) and 5–10% for Alu (Cho et al. 2010; Matsuda et al. 2012). In cancer, this marked hypomethylation of repetitive elements is thought to contribute to widespread genomic instability, which is a hallmark of most malignancies, but the functional importance of relatively small differences in these repetitive elements observed in nonpathologic tissues remains an outstanding question (reviewed by Nelson et al. 2011).

Studies focused on exposure-associated differences in the methylation status of specific candidate genes, as well as more recent epigenome-wide association studies, have commonly found only small effect estimates in regard to differences in methylation by exposure. In general, the differences in methylation observed between groups of exposed versus unexposed individuals, or in relation to some exposure, are generally on the scale of 2–10%, although in some cases even smaller differences have been reported (Table 3). What is striking is that in many cases there is a strong statistical significance (i.e., *p*-values) reported with these small differences suggesting that there is little variability in the measured values. In a number of cases, these differences have been validated in different study populations and even among different ages. This is particularly true for the work that has been done linking maternal smoking during pregnancy and DNA methylation in infant blood, further suggesting the robustness of these relatively small effects (Joubert et al. 2012; Knopik et al. 2012; Lee et al. 2015; Markunas et al. 2014; Richmond et al. 2015).

**Table 3:** Effect sizes of DNA methylation variation from studies of maternal exposures *in utero.*

Exposure	*n*	Magnitude^*a*^	Tissue	Assay/gene	Validation/ replication	Notes	Reference
Maternal smoking	572	–0.04 to 0.07	Peripheral blood	450K array	This study replicated previously identified set of 26 smoking-associated loci	Evaluation of 26 smoking associated loci in 3- to 5-year-old children	Ladd-Acosta et al. 2016
Maternal smoking	6,685	–0.10 to 0.07	Cord blood	450K array	Look up replication in cohorts of older children	> 6,000 smoking-associated loci identified, including 2,965 CpGs corresponding to 2,017 genes not previously related to smoking and methylation in either newborns or adults	Joubert et al. 2016
Maternal smoking	92	–0.02 to 0.1	Peripheral blood	450K array	None	Discovery sample of adolescents with maternal smoking, validated in additional cohorts	Flanagan et al. 2015
Maternal smoking	889	–0.04 to 0.06	Cord blood	450K array	Replication using available EWAS		Markunas et al. 2014
Maternal smoking	800	–0.2 to 0.15	Cord blood	450 K array	None	Some methylation patterns sustained into adolescence	Richmond et al. 2015
Maternal smoking	20	0.04 to 0.09; Overall global hypomethylation	Cord blood	450K array; ELISA	None		Ivorra et al. 2015
Maternal smoking	46	–0.01	Cord blood (mononuclear cells)	Sequenom-*AHRR, GFI1, MYOG1*	None	Found hypomethylation of *AHRR* to 18 months, did not confirm *GFI1*, *MYOG1*	Novakovic et al. 2014
Maternal smoking		–0.28 to 0.18 depending on CpG	Cord blood	450K array	Replication in second cohort	*AHRR* and *CYP1A1* validated	Joubert et al. 2012
Maternal smoking		–0.02 to 0.03	Peripheral blood (5–12 years)	27K array			Breton et al. 2009
Infant toenail Hg	41	0.13 to 0.2 between tertiles	Placenta	450K array	None	Confirmed expression with methylation	Maccani et al. 2015
Maternal toenail Hg	138	0.04 to 0.1 [per log_2_(μg/g Hg)], interaction 0.04 to 0.1	Cord blood	450K array	None	Increase in estimated monocyte proportion with Hg, increase in B-cell proportion in females	Cardenas et al. 2015
First trimester urinary phenols/phthalates	196	–0.35 to –0.4 [per log(mol/L)]	Placenta	PSQ*-H19, IGF2 DMR0, IGF2 DMR2*	None	Interaction with sex	LaRocca et al. 2014
Maternal drinking water As	44	–0.6 to 0.2^*b*^ [per log_10_(μg/L water As)]	Cord blood	450K array	None	Decreases in estimated CD4^+^ T cells, increases in estimated CD8^+^ T cells	Kile et al. 2014
Maternal urinary arsenic	127	–0.01 to 0.03 in boys (per log_2_ increase As); –0.004 to 0.01 in girls (per log_2_ increase As)	Cord blood	450K array	None	More effect in boys than girls	Broberg et al. 2014
Maternal urinary As	134	–0.2 to 0.2 depending on arsenic biomarker (per log increase)	Cord blood	450K array	None	Increase in estimated CD8^+^ T cell	Koestler et al. 2013
Air pollution PM_2.5_	381	0.91% for MT-RNR1, 0.21 P-loop (per interquartile range); Reduction of 15% in mitochondrial content	Placenta	PSQ	None	Mitochondrial DNA	Janssen et al. 2015
Maternal urinary Cd	127	0.3 to 0.4^*c*^	Cord blood	450K array	None	More effect in boys than girls	Kippler et al. 2013
Farm exposure	46	1–2%	Cord blood	PSQ	Replication in 30 additional samples		Michel et al. 2013
Hg, mercury. ^***a***^Magnitude directions are relative to the exposed vs. the unexposed. ^***b***^M-scale. ^***c***^Correlation coefficients.							

One of the most common ways to determine the functional consequence of an observed change in methylation is to study the impact of methylation on gene transcription. Made more powerful by simultaneous extraction and analysis of DNA and RNA from the same cell populations, DNA methylation levels can be correlated with the RNA levels to determine if there is a positive, a negative, or no correlation. In most cases, DNA methylation in gene promoters is negatively associated with transcription, whereas methylation in gene bodies is positively correlated with expression (Ball et al. 2009), consistent with the known effects of DNA methylation on chromatin condensation and transcriptional activity.

Small changes in methylation can have a strong effect on transcriptional activity. Analysis of the imprinted insulin-like growth factor II (*IGF2*) gene in umbilical cord blood determined that for every 1% change in methylation at the *IGF2* differentially methylated region, there was a halving (increased methylation) or doubling (decreased methylation) of *IGF2* transcription (Murphy et al. 2012). This change is equivalent to what would be expected if this gene had a complete loss of imprinted expression. The scale of this change is also equivalent to what is often observed in cancer due to loss of imprinting. Another study examining associations between mercury exposure (measured from toenails) and DNA methylation in placenta as this relates to neurodevelopmental outcomes found over 300 CpGs that had methylation differences greater than ~ 12.5%, comparing tertiles (Maccani et al. 2015). The methylation levels of the CpGs analyzed in *EMID2* were also moderately inversely correlated with transcription (correlation coefficients, –0.33 to –0.45). Study of DNA methylation associated with arsenic exposure in blood also identified correlations between methylation and expression for 28 CpGs, of which about one-third were positively correlated and one-third negatively correlated with expression (Argos et al. 2015). The remainder had multiple gene expression probes associated with each CpG, with the gene probes showing both positive and negative correlations with expression.

It is important to note that beyond the potential functional ramifications for changes in DNA methylation, the covalent nature of this molecular modification and its mitotic heritability provide a means to utilize the particular changes, alone or in combination, as biomarkers of *a*) past exposure, *b*) disease risk, or *c*) for disease detection. DNA methylation-based tests are already in use for detection of colorectal carcinoma (e.g., Cologuard®; Exact Sciences, Madison, WI), and are currently being developed for a number of other types of malignancies. Other methylation changes may be able to predict risk of developing a disease (Cui et al. 2003), information useful for implementation of strategies to reduce risk. Methylation changes may also provide biological documentation of historical exposures or adverse conditions, such as that reported for the individuals subjected to famine conditions *in utero* during the Dutch Hunger Winter in the 1940s in which exposure was associated with small but significant changes in methylation that were detectable in peripheral blood leukocytes six decades past the exposure (Heijmans et al. 2008).

### Genomic Contributions to DNA Methylation Variation

It is increasingly apparent that future investigations in environmental epigenetics will also have to consider genomic context. In a study by Soto-Ramirez et al. (2013), the *IL-4R* SNP rs3024685 carried a significant risk for asthma only when controlled for *IL-4R* methylation. In a study of children ages 2–4 years in Spain, researchers showed that hypomethylation of CpG site in the arachidonate 12-lipoxygenase gene not only correlated with wheezing, but also correlated with the genotype for haplotype-tagging SNP rs312466 (Morales et al. 2012). Genomic variation in the promoter of the nitric oxide synthase (*NOS2*) gene in combination with air pollution exposure affected *iNOS* methylation levels (Salam et al. 2012). Specifically, increased 7-day average PM_2.5_ exposure was associated with lower iNOS methylation, *NOS2* promoter haplotypes were globally associated with *NOS2* promoter methylation, and there was a 3-way interaction among one common promoter haplotype, *iNOS* methylation level, and PM_2.5_ (particulate matter ≤ 2.5 μm) exposure on exhaled nitric oxide levels. A recent study of paraoxonase gene *PON1* demonstrated how one can characterize multiple sources of variability—genetic, epigenetic, and expression—to determine important modulators of candidate susceptibility genes. Using causal mediation analysis, the study provided evidence that DNA methylation mediates the relationship between *PON1_–108_* genotype and *PON1* expression measured by arylesterase activity (Huen et al. 2015).

Another example of the influence of underlying genetic variation was seen in the Brisbane Systems Genomics Study family cohort, which determined that the genetic contribution to CpG methylation state was highly variable and was dependent on degree of heritability. The effect size of such highly heritable *cis*-acting SNPs explained 50–85% of the variation in methylation at these sites (Shah et al. 2014). The importance of incorporating both genetic and environmental covariates in longitudinal study design was illustrated by Shah et al. (2014) in the Lothiah Birth Cohort, in which single nucleotide variation was associated with CpG methylation in 12/37 (32%) of CpG sites that had previously been identified strongly associated with smoking exposures. A further evaluation of the two CpG sites with highest repeatability and heritability found underlying SNP effects that explained 10% of the methylation variation, which was similar to the original effect size of smoking (Shah et al. 2014). In this case, estimates of both genetic and environmental contributions are significantly associated with CpG methylation variation and drift or lack of drift over time.

### Tissue or Cell Type Specific Effects

Most studies of the environmental impact on epigenetics in a children’s health context are using accessible biological samples, including peripheral or cord blood, placenta, or buccal samples. These samples are constituted by a heterogeneous collection of cells. The differences in extent of DNA methylation observed between exposure groups or outcomes thus represent the fraction of the alleles within that given heterogeneous sample which demonstrate methylation. Essentially there is a dilution effect for the magnitude of changes or differences in methylation amongst this sample. To avoid this, one suggestion might be to try and reduce the heterogeneity, by enriching for certain cell populations. For example, in blood, one could focus on a specific lymphocyte subtype, such as CD4^+^ cells, which could be isolated using magnetic bead or FACS (fluorescence-activated cell sorting) technology. Although a desirable approach, there are still some limitations which need to be considered. First is the selection of the cell of interest, which often is not known or which may differ depending on the type of phenotype being interrogated. Second, even technically proficient cell enrichment does not lead to a perfectly homogeneous cell population—even within a given cell type, there are separate clonal outgrowths derived from different stem cell populations—so dilution of the effect may still be an issue. The technical difficulty of this type of enrichment also cannot be overlooked. In blood and most tissues, such purification is really only possible with freshly collected samples, because intact cell membranes and the cell type specific epitopes on those membranes are required for isolation. In addition, although FACS approaches could allow for multiple cell types to be isolated simultaneously, this requires significant expertise and appropriately validated, reproducible, reliable antibodies that can be used to select cell populations. This makes applying such enrichment techniques technically challenging in most existing cohort studies, because these studies are making use of archived samples, no longer able to be subject to such enrichment.

Despite these advances, even in EWAS (epigenome-wide association studies) controlling for cell composition, findings of specific differentially methylated loci or genes associated with exposure or outcomes may still represent cellular composition effects. An example might be activation of specific leukocytes (i.e., NK cells, monocytes) to their active forms. Although these cells may still exhibit similarities in their surface moieties, at the DNA level, methylation may be involved in these final stages of differentiation. If environmental factors drive these differentiation processes, they might be observed as differentially methylated loci. A recent study by Bauer et al. (2015) demonstrated this possibility, identifying a specific *T*-cell subset characterized by hypomethylation of cg19859270, within the *GPR15* gene, a loci that has repeatedly been identified to be hypomethylated amongst smokers. Although this does lead to different interpretations of findings, these findings are nonetheless important, and in fact might provide a better understanding of the functional impact of observed differential methylation.

Although identifying such tissue-specific effects may be important in indicating changes in the cellular landscape related to environmental exposures, there still remains an outstanding question of whether there can be environmentally induced epigenetic changes that could be more broadly identified across tissues. Such findings in humans would parallel those observed in the murine agouti models, where early developmental effects can lead to widespread epigenetic alterations, which in those cases leads to coat color and metabolic effects in the animals (Bernal and Jirtle 2010; Dolinoy et al. 2006, 2007; Jirtle 2014). These effects are specifically observed at regions of hypervariable methylation, known as metastable epialleles, which would represent genomic regions that demonstrate low within-person (across tissue) variability in DNA methylation, but higher between-person variability. These loci would be particularly sensitive to environmental insults during the early cleavage, gastrulation, and initial embryonic stages, allowing for the consistency of the methylation status across various tissues from different embryonic lineages. A recent genome-wide scan using bisulfite sequencing revealed the presence of approximately 100 of these metastable epiallelic regions in the human genome, and found that one in the genomically imprinted *VTRNA2-1* noncoding RNA was environmentally labile, being affected by the nutritional availability during the conception and early gastrulation period in a number of different cohorts examined (Silver et al. 2015). Additional studies focused on these potentially environmentally labile regions are warranted and may provide the opportunity to demonstrate true epigenetic changes linked to environmental exposures experienced during the earliest points of development.

### Epigenome Editing

The development of technologies for locus-specific epigenome editing remains a central challenge in functional genomics, with future applicability to children’s environmental health. Developing these technologies may allow for highly targeted assessments of the functional significance of novel findings of altered DNA methylation or histone post-translational modifications. Many current technologies act globally and cannot target individual loci. For example, pharmaceutical agents, such as azacytidine, are widely used to inhibit DNA methyltransferases, resulting in global hypomethylation in dividing cells (Yang et al. 2010). An advantage of global approaches lies in their well-characterized use as human therapeutics and for basic research in cell lines and animals. Disadvantages, however, include their pleiotropic effects caused by indiscriminate epigenomic activity and propensity to affect biochemical pathways separate from the epigenome.

New methods of locus-specific epigenetic editing have been recently developed that rely upon transgenic technologies. For example, fusions of epigenome-modifying enzymes to programmable DNA-binding proteins hold promise for targeting DNA methylation (Maeder et al. 2013) as well as histone acetylation (Hilton et al. 2015) and epiproteomes (Waldrip et al. 2014) at specific loci; but they have drawbacks, for example, because every zinc-finger domain must be custom evolved to target a specific sequence, and target motifs are size limited. One recent innovation in the field of target specific DNA methylation is the development of a suite of tools, based on the Piwi-interacting RNA (piRNA) system, to accurately induce DNA methylation of targeted loci in adult tissues (work presently being done under NIH grant ES026877; https://directorsblog.nih.gov/tag/pirna/). The major strength in the piRNA approach is that induced changes in DNA methylation will be propagated by endogenous epigenetic maintenance pathways. Thus, piRNA treatment for both laboratory and clinical use will be acute and systemic, rather than chronic with potentially decreasing effectiveness.

## The Future of Environmental Epigenetics in Children’s Health Studies

### Gains from Longitudinal Studies

Although most epigenomic studies have been cross-sectional to date, the prospect of longitudinal studies holds much promise. For example, the first integrative personal ‘omics profiling (iPOP) efforts in 2012 revealed significant dynamic ‘omics changes in peripheral blood mononuclear cells (PBMCs) and serum from one generally healthy individual, demonstrating that these comprehensive molecular portraits reflected real-time physiological states and physiological state changes in this individual (Chen et al. 2012; Chen and Snyder 2013). An important lesson from this personalized medicine proof-of-principle study is that one is her/his best control over time. Different individuals have different baselines, and intrapersonal changes may be masked by interpersonal differences when using case–control design. Mouse models such as the one by Kanzleiter et al. (2015) have also demonstrated longitudinal methylomic differences in skeletal muscle cells in response to exercise training. The authors reported 2,762 differentially methylated genes associated with exercise training, and that ~ 13% of these methylomic differences also were associated with differential expression of the corresponding genes. The majority of the affected genes function in muscle growth and differentiation, as well as in metabolic regulation.

### Moving beyond DNA Methylation

Population-based studies have focused predominantly on DNA methylation as the epigenetic mark of choice. However, other epigenetics marks, including chromatin modifications, microRNAs (miRNAs), and noncoding RNAs warrant further consideration as the technological and economic hurdles of assessing these marks in large numbers decrease.

Chromatin modifications have long been identified as important epigenomic markers involved in diseases and have been associated with multiple diseases such as cancer (Singh et al. 2015; Su et al. 2015), diabetes, and obesity (Schones et al. 2015). Different sequencing methods have been developed to probe high-dimensional chromatin structures (Rao et al. 2014) as well as chromatin-transcription factor interactions (Kellis et al. 2014). All these epigenomic factors may affect downstream gene expression and regulation, which might further lead to changes in physiological states.

In recent years miRNAs have emerged as another epigenetic regulatory mechanism that may play a role in disease onset/pathology by regulating protein interactions. The role of miRNA regulation in cancer is well established. Recently, more studies are emerging showing their association with other diseases, particularly allergic diseases such as asthma and atopic dermatitis (Chen and Qiao 2015; Kan et al. 2015; Knopik et al. 2012; Lv et al. 2014; Omran et al. 2013; Perry et al. 2015; Salam 2014). The majority of these studies have identified miRNA as potential biomarkers (Kan et al. 2015; Li JJ et al. 2015; Lv et al. 2014; Sawant et al. 2015; Simpson et al. 2014). Multiple *in-vitro* and animal studies indicate that miRNA have a role in asthma development and pathogenesis. The 3´ UTR of the asthma susceptibility gene *HLA-G* is targeted by three different miRNAs: miR-148a, miR-148b, and miR-152 (Tan et al. 2007). Multiple miRNAs have been implicated in playing a proinflammatory role in asthma (Kumar et al. 2010; Lu et al. 2009; Mattes et al. 2009; Polikepahad et al. 2010). In a recent study in pediatric asthma patients, Nakano et al. (2013) showed a role for hsa-mir-15a in altering *VEGFa* expression in peripheral CD4 T cells. Pediatric subjects with asthma had lower expression of hsa-mir-15a in their CD4 T cells, which was associated with higher expression of *VEGF-a*. More in-depth mechanistic studies are needed to understand how miRNA can modulate protein expression and thereby affect downstream immune mechanisms in normal and disease conditions. Taken together, these studies show an important role for miRNA regulation in chronic childhood allergic diseases such as asthma and atopic dermatitis, and warrant further investigation into the role of these miRNAs in regulating the immune system.

Hydroxymethylation has recently been shown potentially to carry biological functions, instead of being just an intermediate product during 5-methylcytosine demethylation (Hahn et al. 2014; Shen et al. 2014). DNA hydroxymethylation has been found to be involved in transcription and chromatin regulation (Iurlaro et al. 2013), contributing to olfactory neuron cellular identity (Colquitt et al. 2013) and to monocyte-osteoclast differentiation (de la Rica et al. 2013; Klug et al. 2013), and the loss of 5 hr mC has been reported to be an epigenetic hallmark of melanoma (Lian et al. 2012). Therefore, the DNA hydroxymethylome could well serve as another epigenomic profile that can provide mechanistic insights into health and disease. As with DNA methlyation, measured effect sizes of these alternative epigenetic marks may also be small, and warrant inclusion in the broader discourse about interpretation of such small differences associated with exposures.

### Data Integration

As ’omics data grow, the need for computationally efficient methods of integrating these data sets to better predict disease risk or to better explain biological systems underlying disease has reached a critical juncture. This need is evident in the recent manuscripts published addressing the need for data integration, with various sophisticated bioinformatics strategies proposed to integrate the variety of epigenomic and other “omics” data sets produced by scientists around the world (Génin and Devoto 2015; Gomez-Cabrero et al. 2014; Pineda et al. 2015; Saha et al. 2014; Wachter and Beißbarth 2015; Zierer et al. 2015). In addition, large consortia efforts such as the NIH Roadmap Epigenomics Mapping Consortium, curate data on DNA methylation, mRNA expression, and changes in histones and in chromatin accessibility, annotating these data across a sweeping array of human cell types and creating genome-wide annotation maps. In turn, these maps can be used to produce novel studies of epigenomic changes in development and disease, as well as of the relations among genomic and epigenomic variations (Roadmap Epigenomics Consortium et al. 2015). This type of data warehouse is a valuable tool that can not only inform data integration efforts, particularly from a systems biology perspective, but also inform *in silico* data validation efforts as discussed earlier.

## Conclusion

Our objective in this review was to discuss the state of the science in environmental epigenetics research within the broader context of children’s environmental health. We have presented a review of the technological tools available for assessing epigenetic marks, methods for data analysis and visualization, and methods for functional follow-up of identified loci. We note that a common finding in environmental epigenetics studies is the small magnitudes of effect that result from environmental exposures. Although it is reasonable and necessary that we question the relevance of such small effects, we present examples in which small effects persist and have been replicated across populations and across time. We encourage a critical discourse on the interpretation of such small changes and further research on their functional relevance for children’s health and adult disease susceptibility. It may be the case that we do not find larger effect sizes—not because they do not exist, but rather because such large shifts may be incompatible with continued development.

Children’s environmental health research has made great strides in recent years; yet it is clear that the dynamic nature of the epigenome will require an emphasis on future longitudinal studies in which the epigenome is profiled over time, over changing environmental exposures, and over generations to truly gain a better understanding of the multiple ways in which the epigenome may respond to environmental stimuli. Such longitudinal studies will improve our ability to identify small changes and the consistency of these changes across time and to specific events across development and into adulthood.

## References

[r1] Accomando WP Wiencke JK Houseman EA Nelson HH Kelsey KT 2014 Quantitative reconstruction of leukocyte subsets using DNA methylation. Genome Biol 15 R50, doi:10.1186/gb-2014-15-3-r50 24598480 PMC4053693

[r2] Argos M Chen L Jasmine F Tong L Pierce BL Roy S 2015 Gene-specific differential DNA methylation and chronic arsenic exposure in an epigenome-wide association study of adults in Bangladesh. Environ Health Perspect 123 64 71, doi:10.1289/ehp.1307884 25325195 PMC4286273

[r3] Aryee MJ, Jaffe AE, Corrada-Bravo H, Ladd-Acosta C, Feinberg AP, Hansen KD (2014). Minfi: a flexible and comprehensive Bioconductor package for the analysis of Infinium DNA methylation microarrays.. Bioinformatics.

[r4] Baccarelli A, Wright RO, Bollati V, Tarantini L, Litonjua AA, Suh HH (2009). Rapid DNA methylation changes after exposure to traffic particles.. Am J Respir Crit Care Med.

[r5] Bakulski KM, Feinberg JI, Andrews SV, Yang J, Brown S, McKenney SL (2016). DNA methylation of cord blood cell types: applications for mixed cell birth studies.. Epigenetics.

[r6] Ball MP, Li JB, Gao Y, Lee JH, LeProust EM, Park IH (2009). Targeted and genome-scale strategies reveal gene-body methylation signatures in human cells.. Nat Biotechnol.

[r7] Barker DJ (1988). Childhood causes of adult diseases.. Arch Dis Child.

[r8] Barker DJ (1995). Fetal origins of coronary heart disease.. BMJ.

[r9] Barker DJ, Osmond C (1988). Low birth weight and hypertension.. BMJ.

[r10] Barker DJ, Osmond C, Law CM (1989). The intrauterine and early postnatal origins of cardiovascular disease and chronic bronchitis.. J Epidemiol Community Health.

[r11] Baron RM, Kenny DA (1986). The moderator-mediator variable distinction in social psychological research: conceptual, strategic, and statistical considerations.. J Pers Soc Psychol.

[r12] Bauer M Linsel G Fink B Offenberg K Hahn AM Sack U 2015 A varying T cell subtype explains apparent tobacco smoking induced single CpG hypomethylation in whole blood. Clin Epigenetics 7 81, doi:10.1186/s13148-015-0113-1 26246861 PMC4526203

[r13] Bellamy GJ, Hinchliffe RF, Crawshaw KC, Finn A, Bell F (2000). Total and differential leucocyte counts in infants at 2, 5 and 13 months of age.. Clin Lab Haematol.

[r14] Bernal AJ, Jirtle RL (2010). Epigenomic disruption: the effects of early developmental exposures.. Birth Defects Res A Clin Mol Teratol.

[r15] Binder AM Michels KB 2013 The causal effect of red blood cell folate on genome-wide methylation in cord blood: a Mendelian randomization approach. BMC Bioinformatics 14 353, doi:10.1186/1471-2105-14-353 24305512 PMC3879006

[r16] Bock C, Tomazou EM, Brinkman AB, Müller F, Simmer F, Gu H (2010). Quantitative comparison of genome-wide DNA methylation mapping technologies.. Nat Biotechnol.

[r17] Boyle P Clement K Gu H Smith ZD Ziller M Fostel JL 2012 Gel-free multiplexed reduced representation bisulfite sequencing for large-scale DNA methylation profiling. Genome Biol 13 R92, doi:10.1186/gb-2012-13-10-r92 23034176 PMC3491420

[r18] Breiman L (2001). Random forests.. Mach Learn.

[r19] Breton CV, Byun HM, Wenten M, Pan F, Yang A, Gilliland FD (2009). Prenatal tobacco smoke exposure affects global and gene-specific DNA methylation.. Am J Respir Crit Care Med.

[r20] Broberg K, Ahmed S, Engström K, Hossain MB, Jurkovic Mlakar S, Bottai M (2014). Arsenic exposure in early pregnancy alters genome-wide DNA methylation in cord blood, particularly in boys.. J Dev Orig Health Dis.

[r21] Burris HH, Braun JM, Byun HM, Tarantini L, Mercado A, Wright RJ (2013). Association between birth weight and DNA methylation of *IGF2*, glucocorticoid receptor and repetitive elements LINE-1 and *Alu*.. Epigenomics.

[r22] Burris HH, Rifas-Shiman SL, Baccarelli A, Tarantini L, Boeke CE, Kleinman K (2012). Associations of LINE-1 DNA methylation with preterm birth in a prospective cohort study.. J Dev Orig Health Dis.

[r23] Butcher LM, Beck S (2015). Probe Lasso: a novel method to rope in differentially methylated regions with 450K DNA methylation data.. Methods.

[r24] Cardenas A, Koestler DC, Houseman EA, Jackson BP, Kile ML, Karagas MR (2015). Differential DNA methylation in umbilical cord blood of infants exposed to mercury and arsenic *in utero*.. Epigenetics.

[r25] Chambaz A, Neuvial P, van der Laan MJ (2011). Estimation of a non-parametric variable importance measure of a continuous exposure.. Electronic Journal of Statistics.

[r26] Chen R, Mias GI, Li-Pook-Than J, Jiang L, Lam HY, Chen R (2012). Personal omics profiling reveals dynamic molecular and medical phenotypes.. Cell.

[r27] Chen R, Snyder M (2013). Promise of personalized omics to precision medicine.. Wiley Interdiscip Rev Syst Biol Med.

[r28] Chen Y, Qiao J (2015). Protein-protein interaction network analysis and identifying regulation microRNAs in asthmatic children.. Allergol Immunopathol (Madr).

[r29] Cheng CK, Chan J, Cembrowski GS, van Assendelft OW (2004). Complete blood count reference interval diagrams derived from NHANES III: stratification by age, sex, and race.. Lab Hematol.

[r30] Chipman HA, George EI, McCulloch RE (2010). BART: Bayesian additive regression trees.. Ann Appl Stat.

[r31] Cho YH, Yazici H, Wu HC, Terry MB, Gonzalez K, Qu M (2010). Aberrant promoter hypermethylation and genomic hypomethylation in tumor, adjacent normal tissues and blood from breast cancer patients.. Anticancer Res.

[r32] Colquitt BM, Allen WE, Barnea G, Lomvardas S (2013). Alteration of genic 5-hydroxymethylcytosine patterning in olfactory neurons correlates with changes in gene expression and cell identity.. Proc Natl Acad Sci U S A.

[r33] Cui H, Cruz-Correa M, Giardiello FM, Hutcheon DF, Kafonek DR, Brandenburg S (2003). Loss of *IGF2* imprinting: a potential marker of colorectal cancer risk.. Science.

[r34] Davey Smith G, Hemani G (2014). Mendelian randomization: genetic anchors for causal inference in epidemiological studies.. Hum Mol Genet.

[r35] Davies MN Volta M Pidsley R Lunnon K Dixit A Lovestone S 2012 Functional annotation of the human brain methylome identifies tissue-specific epigenetic variation across brain and blood. Genome Biol 13 R43, doi:10.1186/gb-2012-13-6-r43 22703893 PMC3446315

[r36] De Bustos C Ramos E Young JM Tran RK Menzel U Langford CF 2009 Tissue-specific variation in DNA methylation levels along human chromosome 1. Epigenetics Chromatin 2 7, doi:10.1186/1756-8935-2-7 19505295 PMC2706828

[r37] de la Rica L Rodríguez-Ubreva J García M Islam AB Urquiza JM Hernando H 2013 PU.1 target genes undergo Tet2-coupled demethylation and DNMT3b-mediated methylation in monocyte-to-osteoclast differentiation. Genome Biol 14 R99, doi:10.1186/gb-2013-14-9-r99 24028770 PMC4054781

[r38] Devaney JM, Wang S, Furbert-Harris P, Apprey V, Ittmann M, Wang BD (2015). Genome-wide differentially methylated genes in prostate cancer tissues from African-American and Caucasian men.. Epigenetics.

[r39] Dolinoy DC, Das R, Weidman JR, Jirtle RL (2007). Metastable epialleles, imprinting, and the fetal origins of adult diseases.. Pediatr Res.

[r40] Dolinoy DC Weidman JR Waterland RA Jirtle RL 2006 Maternal genistein alters coat color and protects Avy mouse offspring from obesity by modifying the fetal epigenome. Environ Health Perspect 114 567 572, doi:10.1289/ehp.8700 16581547 PMC1440782

[r41] Du P Zhang X Huang CC Jafari N Kibbe WA Hou L 2010 Comparison of Beta-value and M-value methods for quantifying methylation levels by microarray analysis. BMC Bioinformatics 11 587, doi:10.1186/1471-2105-11-587 21118553 PMC3012676

[r42] Finer S, Mathews C, Lowe R, Smart M, Hillman S, Foo L (2015). Maternal gestational diabetes is associated with genome-wide DNA methylation variation in placenta and cord blood of exposed offspring.. Hum Mol Genet.

[r43] Flanagan JM, Brook MN, Orr N, Tomczyk K, Coulson P, Fletcher O (2015). Temporal stability and determinants of white blood cell DNA methylation in the breakthrough generations study.. Cancer Epidemiol Biomarkers Prev.

[r44] Fortin JP Labbe A Lemire M Zanke BW Hudson TJ Fertig EJ 2014 Functional normalization of 450K methylation array data improves replication in large cancer studies. Genome Biol 15 503, doi:10.1186/s13059-014-0503-2 25599564 PMC4283580

[r45] Friedman JH (1991). Multivariate adaptive regression splines.. Ann Stat.

[r46] Fritz MS, MacKinnon DP (2007). Required sample size to detect the mediated effect.. Psychol Sci.

[r47] Génin E, Devoto M (2015). Integration of omics data in genetic epidemiology.. Hum Hered.

[r48] Gevaert O (2015). MethylMix: an R package for identifying DNA methylation-driven genes.. Bioinformatics.

[r49] Gomez-Cabrero D Abugessaisa I Maier D Teschendorff A Merkenschlager M Gisel A 2014 Data integration in the era of omics: current and future challenges. BMC Syst Biol 8(suppl 2) I1, doi:10.1186/1752-0509-8-S2-I1 PMC4101704 25032990

[r50] Goodrich JM, Sanchez BN, Dolinoy DC, Zhang Z, Hernández-Ávila M, Hu H (2015). Quality control and statistical modeling for environmental epigenetics: a study on *in utero* lead exposure and DNA methylation at birth.. Epigenetics.

[r51] Gruber S, van der Laan MJ (2012). tmle: an R package for targeted maximum likelihood estimation.. J Stat Softw.

[r52] Grunau C, Schattevoy R, Mache N, Rosenthal A (2000). MethTools—a toolbox to visualize and analyze DNA methylation data.. Nucleic Acids Res.

[r53] Hahn MA, Szabó PE, Pfeifer GP (2014). 5-Hydroxymethylcytosine: a stable or transient DNA modification?. Genomics.

[r54] Hales CN, Barker DJ, Clark PM, Cox LJ, Fall C, Osmond C (1991). Fetal and infant growth and impaired glucose tolerance at age 64.. BMJ.

[r55] Hayes AF 2009 Beyond Baron and Kenny: statistical mediation analysis in the new millennium. Commun Monogr 76;408 420

[r56] Heijmans BT, Tobi EW, Stein AD, Putter H, Blauw GJ, Susser ES (2008). Persistent epigenetic differences associated with prenatal exposure to famine in humans.. Proc Natl Acad Sci U S A.

[r57] Heiss JA Brenner H 2015 Between-array normalization for 450K data. Front Genet 6 92, doi:10.3389/fgene.2015.00092 25806048 PMC4354407

[r58] Hilton IB, D’Ippolito AM, Vockley CM, Thakore PI, Crawford GE, Reddy TE (2015). Epigenome editing by a CRISPR-Cas9-based acetyltransferase activates genes from promoters and enhancers.. Nat Biotechnol.

[r59] Hou L, Zhang X, Zheng Y, Wang S, Dou C, Guo L (2014). Altered methylation in tandem repeat element and elemental component levels in inhalable air particles.. Environ Mol Mutagen.

[r60] Houseman EA Accomando WP Koestler DC Christensen BC Marsit CJ Nelson HH 2012 DNA methylation arrays as surrogate measures of cell mixture distribution. BMC Bioinformatics 13 86, doi:10.1186/1471-2105-13-86 22568884 PMC3532182

[r61] Houseman EA, Molitor J, Marsit CJ (2014). Reference-free cell mixture adjustments in analysis of DNA methylation data.. Bioinformatics.

[r62] Hoyo C, Murtha AP, Schildkraut JM, Jirtle RL, Demark-Wahnefried W, Forman MR (2011). Methylation variation at *IGF2* differentially methylated regions and maternal folic acid use before and during pregnancy.. Epigenetics.

[r63] Huen K, Yousefi P, Bradman A, Yan L, Harley KG, Kogut K (2014). Effects of age, sex, and persistent organic pollutants on DNA methylation in children.. Environ Mol Mutagen.

[r64] Huen K, Yousefi P, Street K, Eskenazi B, Holland N (2015). PON1 as a model for integration of genetic, epigenetic, and expression data on candidate susceptibility genes.. Environ Epigenet.

[r65] Irizarry RA, Ladd-Acosta C, Wen B, Wu Z, Montano C, Onyango P (2009). The human colon cancer methylome shows similar hypo- and hypermethylation at conserved tissue-specific CpG island shores.. Nat Genet.

[r66] Issa JP (2014). Aging and epigenetic drift: a vicious cycle.. J Clin Invest.

[r67] Iurlaro M Ficz G Oxley D Raiber EA Bachman M Booth MJ 2013 A screen for hydroxymethylcytosine and formylcytosine binding proteins suggests functions in transcription and chromatin regulation. Genome Biol 14 R119, doi:10.1186/gb-2013-14-10-r119 24156278 PMC4014808

[r68] Ivorra C Fraga MF Bayón GF Fernández AF Garcia-Vicent C Chaves FJ 2015 DNA methylation patterns in newborns exposed to tobacco in utero. J Transl Med 13 25, doi:10.1186/s12967-015-0384-5 25623364 PMC4312439

[r69] Jaffe AE, Murakami P, Lee H, Leek JT, Fallin MD, Feinberg AP (2012). Bump hunting to identify differentially methylated regions in epigenetic epidemiology studies.. Int J Epidemiol.

[r70] Janssen BG, Byun HM, Gyselaers W, Lefebvre W, Baccarelli AA, Nawrot TS (2015). Placental mitochondrial methylation and exposure to airborne particulate matter in the early life environment: an ENVIR*ON*AGE birth cohort study.. Epigenetics.

[r71] Ji H, Ehrlich LI, Seita J, Murakami P, Doi A, Lindau P (2010). Comprehensive methylome map of lineage commitment from haematopoietic progenitors.. Nature.

[r72] Jirtle RL (2014). The Agouti mouse: a biosensor for environmental epigenomics studies investigating the developmental origins of health and disease.. Epigenomics.

[r73] Johnson WE, Li C, Rabinovic A (2007). Adjusting batch effects in microarray expression data using empirical Bayes methods.. Biostatistics.

[r74] Joubert BR, Felix JF, Yousefi P, Bakulski KM, Just AC, Breton C (2016). DNA methylation in newborns and maternal smoking in pregnancy: genome-wide consortium meta-analysis.. Am J Hum Genet.

[r75] Joubert BR Håberg S Nilsen RM Wang X Vollset SE Murphy SK 2012 450K epigenome-wide scan identifies differential DNA methylation in newborns related to maternal smoking during pregnancy. Environ Health Perspect 120 1425 1431, doi:10.1289/ehp.1205412 22851337 PMC3491949

[r76] Kan Q, Ding S, Yang Y, Zhou X (2015). Expression profile of plasma microRNAs in premature infants with respiratory distress syndrome.. Mol Med Rep.

[r77] Kanzleiter T, Jähnert M, Schulze G, Selbig J, Hallahan N, Schwenk RW (2015). Exercise training alters DNA methylation patterns in genes related to muscle growth and differentiation in mice.. Am J Physiol Endocrinol Metab.

[r78] Kellis M, Wold B, Snyder MP, Bernstein BE, Kundaje A, Marinov GK (2014). Defining functional DNA elements in the human genome.. Proc Natl Acad Sci U S A.

[r79] Kile ML Baccarelli A Hoffman E Tarantini L Quamruzzaman Q Rahman M 2012 Prenatal arsenic exposure and DNA methylation in maternal and umbilical cord blood leukocytes. Environ Health Perspect 120 1061 1066, doi:10.1289/ehp.1104173 22466225 PMC3404653

[r80] Kile ML, Houseman EA, Baccarelli A, Quamruzzaman Q, Rahman M, Mostofa G (2014). Effect of prenatal arsenic exposure on DNA methylation and leukocyte subpopulations in cord blood.. Epigenetics.

[r81] Kippler M, Engstrom K, Mlakar SJ, Bottai M, Ahmed S, Hossain MB (2013). Sex-specific effects of early life cadmium exposure on DNA methylation and implications for birth weight.. Epigenetics.

[r82] Klug M Schmidhofer S Gebhard C Andreesen R Rehli M 2013 5-Hydroxymethylcytosine is an essential intermediate of active DNA demethylation processes in primary human monocytes. Genome Biol 14 R46, doi:10.1186/gb-2013-14-5-r46 23705593 PMC4053946

[r83] Knopik VS, Maccani MA, Francazio S, McGeary JE (2012). The epigenetics of maternal cigarette smoking during pregnancy and effects on child development.. Dev Psychopathol.

[r84] Koestler DC Avissar-Whiting M Houseman EA Karagas MR Marsit CJ 2013 Differential DNA methylation in umbilical cord blood of infants exposed to low levels of arsenic *in utero*. Environ Health Perspect 121 971 977, doi:10.1289/ehp.1205925 23757598 PMC3733676

[r85] Kumar M Mabalirajan U Agrawal A Ghosh B 2010 Proinflammatory role of *let-7* miRNAs in experimental asthma? J Biol Chem 285 le19, doi:10.1074/jbc.L110.145698 21097512 PMC2988398

[r86] Ladd-Acosta C, Aryee MJ, Ordway JM, Feinberg AP 2010 Comprehensive high-throughput arrays for relative methylation (CHARM). Curr Protoc Hum Genet Chapter 20:Unit 20.1.1–19, doi:10.1002/0471142905.hg2001s65 PMC6353553 20373514

[r87] Ladd-Acosta C, Shu C, Lee BK, Gidaya N, Singer A, Schieve LA, et al (2016). Presence of an epigenetic signature of prenatal cigarette smoke exposure in childhood.. Environ Res.

[r88] Laird PW (2010). Principles and challenges of genomewide DNA methylation analysis.. Nat Rev Genet.

[r89] Lam LL, Emberly E, Fraser HB, Neumann SM, Chen E, Miller GE (2012). Factors underlying variable DNA methylation in a human community cohort.. Proc Natl Acad Sci U S A.

[r90] LaRocca J, Binder AM, McElrath TF, Michels KB (2014). The impact of first trimester phthalate and phenol exposure on *IGF2/H19* genomic imprinting and birth outcomes.. Environ Res.

[r91] Lazarus J, Mather KA, Armstrong NJ, Song F, Poljak A, Thalamuthu A (2015). DNA methylation in the apolipoprotein-A1 gene is associated with episodic memory performance in healthy older individuals.. J Alzheimers Dis.

[r92] Lee KW Richmond R Hu P French L Shin J Bourdon C 2015 Prenatal exposure to maternal cigarette smoking and DNA methylation: epigenome-wide association in a discovery sample of adolescents and replication in an independent cohort at birth through 17 years of age. Environ Health Perspect 123 193 199, doi:10.1289/ehp.1408614 25325234 PMC4314251

[r93] Leek JT, Johnson WE, Parker HS, Jaffe AE, Storey JD (2012). The sva package for removing batch effects and other unwanted variation in high-throughput experiments.. Bioinformatics.

[r94] Leek JT, Storey JD (2007). Capturing heterogeneity in gene expression studies by surrogate variable analysis.. PLoS Genet.

[r95] Leek JT, Storey JD (2008). A general framework for multiple testing dependence.. Proc Natl Acad Sci U S A.

[r96] Lendle SD, Subbaraman MS, van der Laan MJ (2013). Identification and efficient estimation of the natural direct effect among the untreated.. Biometrics.

[r97] Leon DA, Lithell HO, Vâgerö D, Koupilová I, Mohsen R, Berglund L (1998). Reduced fetal growth rate and increased risk of death from ischaemic heart disease: cohort study of 15 000 Swedish men and women born 1915–29.. BMJ.

[r98] Li JJ, Tay HL, Maltby S, Xiang Y, Eyers F, Hatchwell L (2015). MicroRNA-9 regulates steroid-resistant airway hyperresponsiveness by reducing protein phosphatase 2A activity.. J Allergy Clin Immunol.

[r99] Li Q, Kappil MA, Li A, Dassanayake PS, Darrah TH, Friedman AE (2015). Exploring the associations between microRNA expression profiles and environmental pollutants in human placenta from the National Children’s Study (NCS).. Epigenetics.

[r100] Li SD (2011). Testing mediation using multiple regression and structural equation modeling analyses in secondary data.. Eval Rev.

[r101] Lian CG, Xu Y, Ceol C, Wu F, Larson A, Dresser K (2012). Loss of 5-hydroxymethylcytosine is an epigenetic hallmark of melanoma.. Cell.

[r102] Lisanti S Omar WA Tomaszewski B De Prins S Jacobs G Koppen G 2013 Comparison of methods for quantification of global DNA methylation in human cells and tissues. PLoS One 8 e79044, doi:10.1371/journal.pone.0079044 24260150 PMC3832524

[r103] Liu X, Chen Q, Tsai HJ, Wang G, Hong X, Zhou Y (2014). Maternal preconception body mass index and offspring cord blood DNA methylation: exploration of early life origins of disease.. Environ Mol Mutagen.

[r104] Lokk K Modhukur V Rajashekar B Märtens K Mägi R Kolde R 2014 DNA methylome profiling of human tissues identifies global and tissue-specific methylation patterns. Genome Biol 15 r54, doi:10.1186/gb-2014-15-4-r54 24690455 PMC4053947

[r105] Lowe R, Slodkowicz G, Goldman N, Rakyan VK (2015). The human blood DNA methylome displays a highly distinctive profile compared with other somatic tissues.. Epigenetics.

[r106] Lu TX, Munitz A, Rothenberg ME (2009). MicroRNA-21 is up-regulated in allergic airway inflammation and regulates IL-12p35 expression.. J Immunol.

[r107] Lv Y Qi R Xu J Di Z Zheng H Huo W 2014 Profiling of serum and urinary microRNAs in children with atopic dermatitis. PLoS One 9 e115448, doi:10.1371/journal.pone.0115448 25531302 PMC4274001

[r108] Maccani JZ Koestler DC Lester B Houseman EA Armstrong DA Kelsey KT 2015 Placental DNA methylation related to both infant toenail mercury and adverse neurobehavioral outcomes. Environ Health Perspect 123 723 729, doi:10.1289/ehp.1408561 25748564 PMC4492267

[r109] Maccani JZ, Maccani MA (2015). Altered placental DNA methylation patterns associated with maternal smoking: current perspectives.. Adv Genomics Genet.

[r110] Maeder ML, Angstman JF, Richardson ME, Linder SJ, Cascio VM, Tsai SQ (2013). Targeted DNA demethylation and activation of endogenous genes using programmable TALE-TET1 fusion proteins.. Nat Biotech.

[r111] Maksimovic J Gagnon-Bartsch JA Speed TP Oshlack A 2015 Removing unwanted variation in a differential methylation analysis of Illumina HumanMethylation450 array data. Nucleic Acids Res 43 e106, doi:10.1093/nar/gkv526 25990733 PMC4652745

[r112] Mallona I Díez-Villanueva A Peinado MA 2014 Methylation plotter: a web tool for dynamic visualization of DNA methylation data. Source Code Biol Med 9 11, doi:10.1186/1751-0473-9-11 25260021 PMC4066318

[r113] Markunas CA Xu Z Harlid S Wade PA Lie RT Taylor JA 2014 Identification of DNA methylation changes in newborns related to maternal smoking during pregnancy. Environ Health Perspect 122 1147 1153, doi:10.1289/ehp.1307892 24906187 PMC4181928

[r114] Martin TC Yet I Tsai PC Bell JT 2015 coMET: visualisation of regional epigenome-wide association scan results and DNA co-methylation patterns. BMC Bioinformatics 16 131, doi:10.1186/s12859-015-0568-2 25928765 PMC4422463

[r115] Matsuda Y, Yamashita S, Lee YC, Niwa T, Yoshida T, Gyobu K (2012). Hypomethylation of Alu repetitive elements in esophageal mucosa, and its potential contribution to the epigenetic field for cancerization.. Cancer Causes Control.

[r116] Mattes J, Collison A, Plank M, Phipps S, Foster PS (2009). Antagonism of microRNA-126 suppresses the effector function of T_H_2 cells and the development of allergic airways disease.. Proc Natl Acad Sci U S A.

[r117] Michel S, Busato F, Genuneit J, Pekkanen J, Dalphin JC, Riedler J (2013). Farm exposure and time trends in early childhood may influence DNA methylation in genes related to asthma and allergy.. Allergy.

[r118] Morales E, Bustamante M, Vilahur N, Escaramis G, Montfort M, de Cid R (2012). DNA hypomethylation at *ALOX12* is associated with persistent wheezing in childhood.. Am J Respir Crit Care Med.

[r119] Moran S, Arribas C, Esteller M (2016). Validation of a DNA methylation microarray for 850,000 CpG sites of the human genome enriched in enhancer sequences.. Epigenomics.

[r120] Morris TJ, Beck S (2015). Analysis pipelines and packages for Infinium HumanMethylation450 BeadChip (450k) data.. Methods.

[r121] Morris TJ, Butcher LM, Feber A, Teschendorff AE, Chakravarthy AR, Wojdacz TK (2014). ChAMP: 450k Chip Analysis Methylation Pipeline.. Bioinformatics.

[r122] Murphy SK, Adigun A, Huang Z, Overcash F, Wang F, Jirtle RL (2012). Gender-specific methylation differences in relation to prenatal exposure to cigarette smoke.. Gene.

[r123] Nahar MS, Kim JH, Sartor MA, Dolinoy DC (2014). Bisphenol A-associated alterations in the expression and epigenetic regulation of genes encoding xenobiotic metabolizing enzymes in human fetal liver.. Environ Mol Mutagen.

[r124] Nakano T, Inoue Y, Shimojo N, Yamaide F, Morita Y, Arima T, et al (2013). Lower levels of hsa-mir-15a, which decreases *VEGFA*, in the CD4^+^ T cells of pediatric patients with asthma.. J Allergy Clin Immunol.

[r125] Nelson HH Marsit CJ Kelsey KT 2011 Global methylation in exposure biology and translational medical science. Environ Health Perspect 119 1528 1533, doi:10.1289/ehp.1103423 21669556 PMC3226501

[r126] Novakovic B, Ryan J, Pereira N, Boughton B, Craig JM, Saffery R (2014). Postnatal stability, tissue, and time specific effects of AHRR methylation change in response to maternal smoking in pregnancy.. Epigenetics.

[r127] Olden K, Lin YS, Gruber D, Sonawane B (2014). Epigenome: biosensor of cumulative exposure to chemical and nonchemical stressors related to environmental justice.. Am J Public Health.

[r128] Oliver VF, Franchina M, Jaffe AE, Branham KE, Othman M, Heckenlively JR (2013). Hypomethylation of the *IL17RC* promoter in peripheral blood leukocytes is not a hallmark of age-related macular degeneration.. Cell Rep.

[r129] Omran A Elimam D Yin F 2013 MicroRNAs: new insights into chronic childhood diseases. Biomed Res Int 2013 291826, doi:10.1155/2013/291826 23878802 PMC3710618

[r130] Paquette AG, Lester BM, Lesseur C, Armstrong DA, Guerin DJ, Appleton AA (2015). Placental epigenetic patterning of glucocorticoid response genes is associated with infant neurodevelopment.. Epigenomics.

[r131] Pedersen BS, Schwartz DA, Yang IV, Kechris KJ (2012). Comb-p: software for combining, analyzing, grouping and correcting spatially correlated *P*-values.. Bioinformatics.

[r132] Perry MM, Adcock IM, Chung KF (2015). Role of microRNAs in allergic asthma: present and future.. Curr Opin Allergy Clin Immunol.

[r133] Peters TJ Buckley MJ Statham AL Pidsley R Samaras K Lord RV 2015 *De novo* identification of differentially methylated regions in the human genome. Epigenetics Chromatin 8 6, doi:10.1186/1756-8935-8-6 25972926 PMC4429355

[r134] Petersen M, Schwab J, Gruber S, Blaser N, Schomaker M, van der Laan M (2014). Targeted maximum likelihood estimation for dynamic and static longitudinal marginal structural working models.. J Causal Inference.

[r135] Petersen ML, Sinisi SE, van der Laan MJ (2006). Estimation of direct causal effects.. Epidemiology.

[r136] Pidsley R Wong CCY Volta M Lunnon K Mill J Schalkwyk LC 2013 A data-driven approach to preprocessing Illumina 450K methylation array data. BMC Genomics 14 293, doi:10.1186/1471-2164-14-293 23631413 PMC3769145

[r137] Pineda S Real FX Kogevinas M Carrato A Chanock SJ Malats N 2015 Integration analysis of three omics data using penalized regression methods: an application to bladder cancer. PLoS Genet 11 e1005689, doi:10.1371/journal.pgen.1005689 26646822 PMC4672920

[r138] Plongthongkum N, Diep DH, Zhang K (2014). Advances in the profiling of DNA modifications: cytosine methylation and beyond.. Nat Rev Genet.

[r139] Polikepahad S, Knight JM, Naghavi AO, Oplt T, Creighton CJ, Shaw C (2010). Proinflammatory role for *let-7* microRNAs in experimental asthma.. J Biol Chem.

[r140] Rao SS, Huntley MH, Durand NC, Stamenova EK, Bochkov ID, Robinson JT (2014). A 3D map of the human genome at kilobase resolution reveals principles of chromatin looping.. Cell.

[r141] Reinius LE Acevedo N Joerink M Pershagen G Dahlén SE Greco D 2012 Differential DNA methylation in purified human blood cells: implications for cell lineage and studies on disease susceptibility. PLoS One 7 e41361, doi:10.1371/journal.pone.0041361 22848472 PMC3405143

[r142] Relton CL, Davey Smith G (2012). Two-step epigenetic Mendelian randomization: a strategy for establishing the causal role of epigenetic processes in pathways to disease.. Int J Epidemiol.

[r143] Relton CL, Davey Smith G (2015). Mendelian randomization: applications and limitations in epigenetic studies.. Epigenomics.

[r144] Richmond RC, Sharp GC, Ward ME, Fraser A, Lyttleton O, McArdle WL (2016). DNA methylation and BMI: investigating identified methylation sites at *HIF3A* in a causal framework.. Diabetes.

[r145] Richmond RC, Simpkin AJ, Woodward G, Gaunt TR, Lyttleton O, McArdle WL (2015). Prenatal exposure to maternal smoking and offspring DNA methylation across the lifecourse: findings from the Avon Longitudinal Study of Parents and Children (ALSPAC).. Hum Mol Genet.

[r146] Roadmap Epigenomics Consortium, Kundaje A, Meuleman W, Ernst J, Bilenky M, Yen A, et al (2015). Integrative analysis of 111 reference human epigenomes.. Nature.

[r147] Robertson KD (2005). DNA methylation and human disease.. Nat Rev Genet.

[r148] Robinson MD Kahraman A Law CW Lindsay H Nowicka M Weber LM 2014 Statistical methods for detecting differentially methylated loci and regions. Front Genet 5 324, doi:10.3389/fgene.2014.00324 25278959 PMC4165320

[r149] Roessler J Ammerpohl O Gutwein J Hasemeier B Anwar SL Kreipe H 2012 Quantitative cross-validation and content analysis of the 450k DNA methylation array from Illumina, Inc. BMC Res Notes 5 210, doi:10.1186/1756-0500-5-210 22546179 PMC3420245

[r150] Saha R, Chowdhury A, Maranas CD (2014). Recent advances in the reconstruction of metabolic models and integration of omics data.. Curr Opin Biotechnol.

[r151] Salam MT (2014). Asthma epigenetics.. Adv Exp Med Biol.

[r152] Salam MT, Byun HM, Lurmann F, Breton CV, Wang X, Eckel SP, et al (2012). Genetic and epigenetic variations in inducible nitric oxide synthase promoter, particulate pollution, and exhaled nitric oxide levels in children.. J Allergy Clin Immunol.

[r153] Sawant DV, Yao W, Wright Z, Sawyers C, Tepper RS, Gupta SK (2015). Serum microRNA-21 as a biomarker for allergic inflammatory disease in children.. Microrna.

[r154] Schones DE, Leung A, Natarajan R (2015). Chromatin modifications associated with diabetes and obesity.. Arterioscler Thromb Vasc Biol.

[r155] Schones DE, Zhao K (2008). Genome-wide approaches to studying chromatin modifications.. Nat Rev Genet.

[r156] Schroeder DI, LaSalle JM (2013). How has the study of the human placenta aided our understanding of partially methylated genes?. Epigenomics.

[r157] Shah S, McRae AF, Marioni RE, Harris SE, Gibson J, Henders AK (2014). Genetic and environmental exposures constrain epigenetic drift over the human life course.. Genome Res.

[r158] Shen L, Song CX, He C, Zhang Y (2014). Mechanism and function of oxidative reversal of DNA and RNA methylation.. Annu Rev Biochem.

[r159] Silver MJ Kessler NJ Hennig BJ Dominguez-Salas P Laritsky E Baker MS 2015 Independent genomewide screens identify the tumor suppressor *VTRNA2-1* as a human epiallele responsive to periconceptional environment. Genome Biol 16 118, doi:10.1186/s13059-015-0660-y 26062908 PMC4464629

[r160] Simpson LJ, Patel S, Bhakta NR, Choy DF, Brightbill HD, Ren X (2014). A microRNA upregulated in asthma airway T cells promotes T_H_2 cytokine production.. Nat Immunol.

[r161] Singh V, Singh LC, Singh AP, Sharma J, Borthakur BB, Debnath A (2015). Status of epigenetic chromatin modification enzymes and esophageal squamous cell carcinoma risk in northeast Indian population.. Am J Cancer Res.

[r162] Smyth GK (2005). limma: linear models for microarray data. In: *Bioinformatics and Computational Biology Solutions Using R and Bioconductor*. Gentleman R, Carey V, Huber W, Irizarry RA, Dudoit S, eds..

[r163] Sofer T, Schifano ED, Hoppin JA, Hou L, Baccarelli AA (2013). A-clustering: a novel method for the detection of co-regulated methylation regions, and regions associated with exposure.. Bioinformatics.

[r164] Soto-Ramirez N Arshad SH Holloway JW Zhang H Schauberger E Ewart S 2013 The interaction of genetic variants and DNA methylation of the interleukin-4 receptor gene increase the risk of asthma at age 18 years. Clin Epigenetics 5 1, doi:10.1186/1868-7083-5-1 23286427 PMC3544634

[r165] Soubry A, Murphy SK, Wang F, Huang Z, Vidal AC, Fuemmeler BF (2015). Newborns of obese parents have altered DNA methylation patterns at imprinted genes.. Int J Obes (Lond).

[r166] Soubry A Schildkraut JM Murtha A Wang F Huang Z Bernal A 2013 Paternal obesity is associated with *IGF2* hypomethylation in newborns: results from a Newborn Epigenetics Study (NEST) cohort. BMC Med 11 29, doi:10.1186/1741-7015-11-29 23388414 PMC3584733

[r167] Steegers-Theunissen RP Obermann-Borst SA Kremer D Lindemans J Siebel C Steegers EA 2009 Periconceptional maternal folic acid use of 400 μg per day is related to increased methylation of the *IGF2* gene in the very young child. PLoS One 4 e7845, doi:10.1371/journal.pone.0007845 19924280 PMC2773848

[r168] Stricker I, Tzivras D, Nambiar S, Wulf J, Liffers ST, Vogt M (2012). Site- and grade-specific diversity of *LINE1* methylation pattern in gastroenteropancreatic neuroendocrine tumours.. Anticancer Res.

[r169] Su Y, Subedee A, Bloushtain-Qimron N, Savova V, Krzystanek M, Li L (2015). Somatic cell fusions reveal extensive heterogeneity in basal-like breast cancer.. Cell Rep.

[r170] Tan Z, Randall G, Fan J, Camoretti-Mercado B, Brockman-Schneider R, Pan L (2007). Allele-specific targeting of microRNAs to *HLA-G* and risk of asthma.. Am J Hum Genet.

[r171] Tang WW, Dietmann S, Irie N, Leitch HG, Floros VI, Bradshaw CR (2015). A unique gene regulatory network resets the human germline epigenome for development.. Cell.

[r172] Tarantini L, Bonzini M, Tripodi A, Angelici L, Nordio F, Cantone L (2013). Blood hypomethylation of inflammatory genes mediates the effects of metal-rich airborne pollutants on blood coagulation.. Occup Environ Med.

[r173] Teschendorff AE, Zhuang J, Widschwendter M (2011). Independent surrogate variable analysis to deconvolve confounding factors in large-scale microarray profiling studies.. Bioinformatics.

[r174] Thompson RF, Suzuki M, Lau KW, Greally JM (2009). A pipeline for the quantitative analysis of CG dinucleotide methylation using mass spectrometry.. Bioinformatics.

[r175] Tost J, Gut IG (2007). DNA methylation analysis by pyrosequencing.. Nat Protoc.

[r176] Tuglus C van der Laan MJ 2011 Repeated measures semiparametric regression using targeted maximum likelihood methodology with application to transcription factor activity discovery. Stat Appl Genet Mol Biol 10:Article 2, doi:10.2202/1544-6115.1553 PMC3122882 21291412

[r177] van der Laan MJ 2010a Targeted maximum likelihood based causal inference: part I. Int J Biostat 6:Article 2, doi:10.2202/1557-4679.1211 PMC3126670 21969992

[r178] van der Laan MJ 2010b Targeted maximum likelihood based causal inference: part II. Int J Biostat 6:Article 3, doi:10.2202/1557-4679.1241 PMC3126672 21731531

[r179] van der Laan MJ, Dudoit S (2003). Unified Cross-Validation Methodology for Selection among Estimators and a General Cross-Validated Adaptive Epsilon-Net Estimator: Finite Sample Oracle Inequalities and Examples.. http://biostats.bepress.com/ucbbiostat/paper130.

[r180] van der Laan MJ, Gruber S (2011). Targeted Minimum Loss Based Estimation of an Intervention Specific Mean Outcome.. http://biostats.bepress.com/ucbbiostat/paper290.

[r181] van der Laan MJ Polley EC Hubbard AE 2007 Super learner. Stat Appl Genet Mol Biol 6:Article 25, doi:10.2202/1544-6115.1309 17910531

[r182] van der Laan MJ, Rose S (2011). *Targeted Learning: Causal Inference for Observational and Experimental Data*..

[r183] van der Laan MJ, Rose S, Gruber S (2009). Readings in Targeted Maximum Likelihood Estimation.. http://biostats.bepress.com/ucbbiostat/paper254.

[r184] van der Laan MJ, Rubin D (2006). Targeted Maximum Likelihood Learning.. http://biostats.bepress.com/ucbbiostat/paper213.

[r185] van der Vaart AW, Dudoit S, van der Laan MJ (2006). Oracle inequalities for multi-fold cross-validation.. J Stat Decisions.

[r186] Vidal AC, Benjamin Neelon SE, Liu Y, Tuli AM, Fuemmeler BF, Hoyo C (2014). Maternal stress, preterm birth, and DNA methylation at imprint regulatory sequences in humans.. Genet Epigenet.

[r187] Vidal AC, Murphy SK, Murtha AP, Schildkraut JM, Soubry A, Huang Z (2013). Associations between antibiotic exposure during pregnancy, birth weight and aberrant methylation at imprinted genes among offspring.. Int J Obes (Lond).

[r188] Vilahur N, Bustamante M, Byun HM, Fernandez MF, Santa Marina L, Basterrechea M (2014). Prenatal exposure to mixtures of xenoestrogens and repetitive element DNA methylation changes in human placenta.. Environ Int.

[r189] Wachter A, Beißbarth T (2015). pwOmics: an R package for pathway-based integration of time-series omics data using public database knowledge.. Bioinformatics.

[r190] Waldrip ZJ, Byrum SD, Storey AJ, Gao J, Byrd AK, Mackintosh SG (2014). A CRISPR-based approach for proteomic analysis of a single genomic locus.. Epigenetics.

[r191] Wang D, Yan L, Hu Q, Sucheston LE, Higgins MJ, Ambrosone CB (2012). IMA: an R package for high-throughput analysis of Illumina’s 450K Infinium methylation data.. Bioinformatics.

[r192] Wang H, Rose S, van der Laan MJ (2011). Finding quantitative trait loci genes with collaborative targeted maximum likelihood learning.. Stat Probab Lett.

[r193] Wei L, Liu B, Tuo J, Shen D, Chen P, Li Z (2012). Hypomethylation of the *IL17RC* promoter associates with age-related macular degeneration.. Cell Rep.

[r194] Wu D Gu J Zhang MQ 2013 FastDMA: an Infinium HumanMethylation450 Beadchip analyzer. PLoS One 8 e74275, doi:10.1371/journal.pone.0074275 24040221 PMC3764200

[r195] Yang X, Lay F, Han H, Jones PA (2010). Targeting DNA methylation for epigenetic therapy.. Trends Pharmacol Sci.

[r196] Yousefi P Huen K Davé V Barcellos L Eskenazi B Holland N 2015a Sex differences in DNA methylation assessed by 450 K BeadChip in newborns. BMC Genomics 16 911, doi:10.1186/s12864-015-2034-y 26553366 PMC4640166

[r197] Yousefi P, Huen K, Quach H, Motwani G, Hubbard A, Eskenazi B (2015b). Estimation of blood cellular heterogeneity in newborns and children for epigenome-wide association studies.. Environ Mol Mutagen.

[r198] Yuan T Jiao Y de Jong S Ophoff RA Beck S Teschendorff AE 2015 An integrative multi-scale analysis of the dynamic DNA methylation landscape in aging. PLoS Genet 11 e1004996, doi:10.1371/journal.pgen.1004996 25692570 PMC4334892

[r199] Zackay A Steinhoff C 2010 MethVisual – visualization and exploratory statistical analysis of DNA methylation profiles from bisulfite sequencing. BMC Res Notes 3 337, doi:10.1186/1756-0500-3-337 21159174 PMC3012040

[r200] Zeger SL, Liang KY, Albert PS (1988). Models for longitudinal data: a generalized estimating equation approach.. Biometrics.

[r201] Zhang YJ, Wu HC, Yazici H, Yu MW, Lee PH, Santella RM (2012). Global hypomethylation in hepatocellular carcinoma and its relationship to aflatoxin B(1) exposure.. World J Hepatol.

[r202] Zierer J, Menni C, Kastenmüller G, Spector TD (2015). Integration of ‘omics’ data in aging research: from biomarkers to systems biology.. Aging Cell.

[r203] Zou J, Lippert C, Heckerman D, Aryee M, Listgarten J (2014). Epigenome-wide association studies without the need for cell-type composition.. Nat Methods.

